# CO_2_ Adsorption on Variably Hydrated Cation-Exchanged Montmorillonite-Rich Clays

**DOI:** 10.1021/acs.jpcc.4c07731

**Published:** 2025-03-26

**Authors:** Niels Mendel, Diana Sîreţanu, Igor Sîreţanu, Derk W. F. Wim Brilman, Frieder Mugele

**Affiliations:** † Physics of Complex Fluids, Faculty of Science and Technology, MESA+ Institute for Nanotechnology, University of Twente, P.O. Box 217, 7500 AE Enschede, The Netherlands; ‡ Sustainable Process Technology, Faculty of Science and Technology, University of Twente, P.O. Box 217, 7500 AE Enschede, The Netherlands

## Abstract

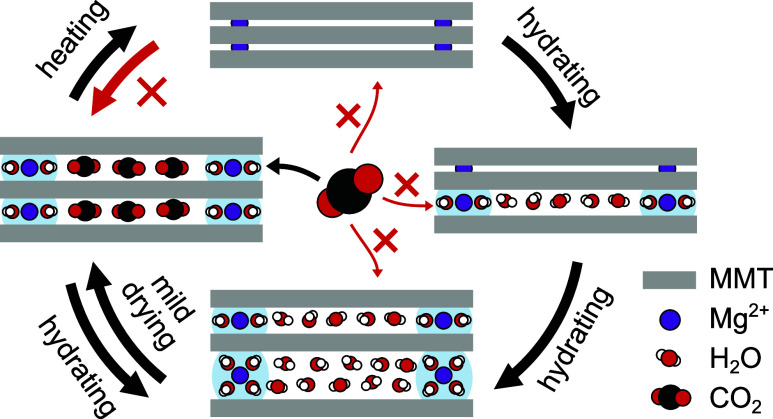

Layered swelling clay minerals like montmorillonite (MMT) can competitively and synergistically adsorb CO_2_ and H_2_O in their interlayer galleries. This work examines how different interlayer cations, relative humidity levels (and amount of cosorbed H_2_O), and (de)hydration history affect CO_2_ adsorption on MMT and MMT-rich bentonite at near-ambient pressure and temperature. For CO_2_ to be adsorbed, the MMT requires either large (e.g., Cs^+^) or hydrated interlayer cations to provide a sufficiently wide interlayer gallery, and must not have too much H_2_O adsorbed competitively with CO_2_. Na-MMT and initially anhydrous Mg- and Ca-MMT studied under increasing relative humidity conditions adsorb little CO_2_. However, Mg- and Ca-MMT can effectively adsorb CO_2_ if first hydrated and then mildly dried such that the cations remain hydrated while the competitively adsorbed excess H_2_O is removed. Because of their high stability and the favorable shape of their CO_2_ adsorption isotherms, low-cost (near-)natural Mg- and Ca-bentonite can be used for (cyclic) CO_2_ adsorption and separation purposes, similar to the more expensive Cs-bentonite.

## 1 Introduction

Swelling layered silicates, known as smectites, can sorb significant amounts of molecules, including H_2_O and CO_2_. Their adsorption capacity is located primarily in the interlayer galleries, provided that these are accessible to the molecules to be sorbed. The general motivation of the present study arises from our goal to use smectites as a sorbent to separate CO_2_ from mixed gas streams under near-ambient conditions.^1,2^ However, the ability of smectites to sorb these molecules is actually significant across a range of conditions, e.g., from high-pressure geological gas storage and carbon sequestration^3−9^ to the low-pressure dynamic exchange of CO_2_ between smectite-bearing soil and the atmosphere.^10^

**Figure 1:**
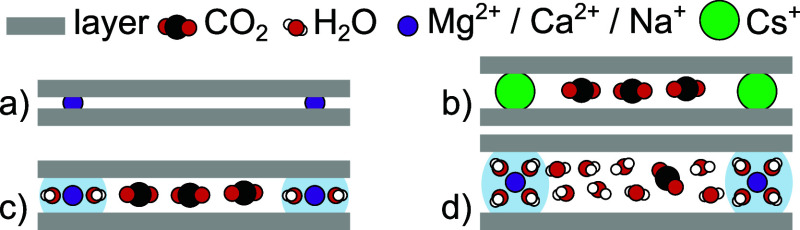
Synergistic and competitive effects for the adsorption of H_2_O and CO_2_. (a) CO_2_ does not adsorb in the interlayer galleries of anhydrous Mg-, Ca-, and Na-smectites, whereas (b) it can adsorb in the interlayer galleries of anhydrous Cs-smectite. (c) Little H_2_O can open the interlayer galleries to permit CO_2_ adsorption therein, whereas (d) excess H_2_O outcompetes CO_2_ adsorption.

Previous studies demonstrated that the adsorption capacity of smectites for CO_2_ and other nonpolar molecules can be varied by tuning the height of their interlayer galleries. This is achieved by exchanging the naturally occurring interlayer cations for others of different sizes (Table S1 and, e.g., refs (11–16)). If the interlayer galleries are too narrow, the molecules cannot enter and hence the sorption capacity is low. This is the case for dry CO_2_ sorption on anhydrous smectites with small cations like Mg^2+^, Ca^2+^, and Na^+^ (Figure 1a).^1,6,9,17−27^ If the interlayer gallery height exceeds the size of the molecule to be sorbed, the molecules can enter the interlayer galleries and hence the sorption capacity of the material is high. Therefore, the sorption capacity for nonpolar gases generally increases with decreasing size of the sorbing gas molecule and/or increasing size of the interlayer cations, e.g., refs (1,11,22,23,25–33). Cs^+^ or larger organic cations are particularly suitable to achieve high sorption capacities for dry CO_2_ under near-ambient conditions (Figure 1b).^1,25,27^

In the present study, we build on the previous work and explore essentially three new directions. First, we investigate the competitive adsorption of CO_2_ and H_2_O. This is particularly important because many mixed gas streams of interest contain finite amounts of humidity and the polar H_2_O molecule is generally known to interact more strongly with smectites than the nonpolar CO_2_ molecule (Figure 1d).^5,9,20,21,26,34−37^ Second, we explore the possibility to replace the Cs^+^ by cheaper and more abundant cations like Mg^2+^, Ca^2+^, and Na^+^. While these bare cations are too small to enable CO_2_ entry, in particular the divalent cations are known to be strongly hydrated even at low relative humidity (RH).^13−16,38,39^ This hydration increases their effective diameter and thereby the interlayer gallery height, thus making it possible for CO_2_ to enter in the presence of low amounts of cosorbed H_2_O (Figure 1c), as demonstrated before under (primarily) high-pressure conditions.^3,5,6,9,17−19,21,24−26,40^ Third, we replace the expensive research grade montmorillonite (MMT) from previous studies by much cheaper commercial (MMT-rich) bentonite.

As we will see, the first two points lead to a dual role of H_2_O in CO_2_ sorption. On the one hand, the hydration of cations is essential to maintain an adequate interlayer gallery height for CO_2_ entry. On the other hand, excess H_2_O competes with CO_2_ for the available sorption space. Optimum CO_2_ sorption is achieved by (i) a suitable pretreatment of the clay that involves dedicated hydration and (mild) drying steps that result from the specific hysteretic nature of H_2_O sorption, ultimately to remove the competitively adsorbed H_2_O while maintaining the relatively stable cation hydration shell, and (ii) overall low H_2_O content in the gas stream from which the CO_2_ is to be sorbed.

## 2 Experimental Methods

### 2.1 Materials

Commercial MMT-rich Na-activated bentonite (Cebogel QSE) was purchased from Eijkelkamp Soil & Water. This bentonite has similar characteristics as research grade MMT SWy (Clay Mineral Society), but with an (assumed passive) impurity fraction of ∼28% based on the difference in CO_2_ adsorption between anhydrous Cs-MMT^27^ and Cs-bentonite.^1^ Therefore, we assume here that the bentonite contains 72% MMT with the stoichiometry of MMT SWy,^41^ i.e., with 0.6 M^+^ or 0.3 M^2+^ per O_20_(OH)_4_ structural unit. The interlayer cations were exchanged for Mg^2+^ or Ca^2+^ by suspending the bentonite, which was first loaded into a dialysis tube (SnakeSkin, 3.5 kDa MCOW), in a solution of MgCl_2_ or CaCl_2_ (hexa- and dihydrate; Sigma-Aldrich; ≥99%) at room temperature for at least 4 weeks. The exchange suspension contained, per gram bentonite, 10 mL Milli-Q water and 3.2 mmol divalent cations. This corresponds to ∼8 times the cation exchange capacity of MMT SWy.^41^ Excess salt was washed off the cation-exchanged bentonites by resuspending the dialysis tube in fresh Milli-Q water six times. The supernatant conductivity remained below 100 μS cm^–1^ during the final washing cycle. Subsequently, the bentonites were dried in an oven at 60 °C and ground manually to a powder using a mortar and pestle. The Na-bentonite is the ground as-received bentonite material and the preparation procedure for Cs-bentonite is detailed in ref (1). Experiments using the thermogravimetric analyzer (see below) used samples of the cation-exchanged MMT SWy (hereafter referred to as MMT) instead. These samples were prepared similarly, as is detailed in ref (27). In our previous works involving the same bentonite and MMT source clays exchanged with these and/or various other cations, refs (1,27, and 32), we demonstrated (i) a large degree of cation exchange for the protocol above or similar, and (ii) using low-temperature N_2_ sorption measurements under anhydrous conditions that the cation exchange barely affects the external surface area of the clays, while the exchange with larger cations (i.e., Cs^+^ and larger) can facilitate adsorption of N_2_ in the interlayer galleries.

### 2.2 H_2_O and CO_2_ Sorption

The sorption of H_2_O, the adsorption of CO_2_, and the stability of the prehydrated samples under dry N_2_ purge, all at room temperature and atmospheric pressure, were measured gravimetrically on an analytical balance (Adam Equipment Nimbus NBL 254i; continuous data-acquisition with a sampling frequency of ∼1 Hz). To this end, the scale platform was enclosed by a small plastic container (*V* ≈ 0.3 L) in which the relative humidity (RH) was controlled with a home-built humidistat^42^ via the inlet gas (flow rate: 2 L min^–1^), see Figure S1. The inlet gas could furthermore be switched between N_2_ and CO_2_ (Grade 4.5; Linde Gas). The RH inside the container could be varied between ∼0% and ∼80% and was monitored using a Sensirion SHT-85 humidity sensor. The balance was furthermore enclosed by a larger plastic container that was continuously purged with dry N_2_ to further reduce the atmospheric water content under the driest conditions. While the humidity sensor displayed RH down to 0.0%, its accuracy is around ±1.5% pt and it is conceivable that the actual RH in the container then was not absolute zero. Two types of experiments were conducted using this setup.

First, the H_2_O adsorption and desorption isotherms and the corresponding (i.e., at each RH along the H_2_O adsorption and desorption branches) CO_2_ adsorption were determined on bentonite samples with a typical mass of 3.3–4.1 g that were placed in a glass Petri dish on the scale platform. Prior to each experiment, the samples were preconditioned by either (i) in situ prehumidification at ∼80% RH for ≥18 h followed by in situ predrying under dry N_2_ purge at room temperature for >40 h, or (ii) ex situ predrying at 150 or 200 °C in an oven without gas purge for 24 h. The H_2_O sorption isotherms (under N_2_ purge) were then determined by first the stepwise increase of the RH from ∼0% to ∼80%, and consecutively the stepwise decrease back to ∼0% RH. At each RH, the sample was equilibrated for ≥18 h, until apparent equilibrium. The mass difference between the equilibrated sample and the dry sample was regarded as the H_2_O sorption. This H_2_O sorption was corrected for drift induced by the variable RH environment. The dry sample mass and amount of retained H_2_O were determined after the adsorption–desorption experiment by (post)drying the sample at 200 °C in an oven without gas purge for 24 h. The CO_2_ adsorption at the given RH was measured (after H_2_O equilibrium was attained) by switching the inlet gas from N_2_ to CO_2_ for 15 min and determined from the subsequent sample mass increase. A calibration experiment with an empty Petri dish was subtracted. The typical (apparent) equilibration time of CO_2_ is a few minutes (i.e., ≪15 min) and much shorter than that of H_2_O.

Second, to test the long-term stability of adsorbed H_2_O under dry N_2_ purge conditions, in particular the effect thereof on the CO_2_ adsorption, a bentonite sample with a typical mass of 3.5–3.9 g was placed in a glass Petri dish on the scale platform and prehydrated in situ at ∼80% RH for ≥18 h. At the start of the experiment, the target RH (under N_2_ purge) was set to 0%. The CO_2_ adsorption as a function of drying time was determined (regularly) following the procedure detailed above, i.e., the switching between CO_2_ and N_2_. During the first few hours of the experiment, the CO_2_ adsorption is underestimated due to the then significant simultaneous desorption of H_2_O.

### 2.3 Thermogravimetric Analyzer

A Netzsch STA449 F3 Jupiter thermal gravimetric analyzer (TGA) with a controlled mixed flow of dry N_2_ and CO_2_ was used to assess the desorption of H_2_O and the adsorption and desorption (isotherms) of CO_2_ on the MMT samples. The total gas flow rate was always 100 mL min^–1^. Prior to each experiment, the samples were prehumidified ex situ by storing an individual vial filled with the MMT sample in a sealed bottle containing a saturated salt solution of KNO_3_ (∼94% RH at 20 °C)^43^ or liquid H_2_O (100% RH) for at least 2 weeks.^44^ The samples were then transferred rapidly to an Al_2_O_3_ crucible that was placed in the TGA. The thermal desorption of H_2_O in the absence of CO_2_ was measured on a sample with a typical mass of 18–20 mg (prehydrated at ∼94% RH) subjected to a heating rate of 1 K min^–1^ under dry N_2_ flow to 300 °C. The CO_2_ adsorption (isotherms) on variably hydrated samples were measured on a sample (∼20–30 mg; prehydrated at 100% RH) subjected to various protocols that were based on CO_2_ adsorption studies on variably hydrated Metal–Organic Frameworks.^45^ The exact protocol used is detailed per set of experiments and involved steps of (i) N_2_ purging at 30 °C (or 90 or 150 °C) to remove of weakly (or strongly) adsorbed H_2_O; (ii) (stepwise) increase of the input gas CO_2_ fraction (0% to 80%) at 30 °C to determine CO_2_ adsorption (isotherm); and (iii) sample heating to 250 or 300 °C under 100% N_2_ to determine the dry sample mass. For all experiments, a calibration measurement with an empty crucible was subtracted. The mass difference between the sample in pure N_2_ and in the mixture of N_2_ and CO_2_ was regarded the CO_2_ adsorption at that partial pressure, under the assumption that H_2_O was not displaced by CO_2_.

### 2.4 Adsorption Isotherms

The CO_2_ adsorption isotherms were measured using a home-built Sieverts apparatus.^1,27^ The sample chamber was loaded with ∼6.5 g of the bentonite powder and immersed in a Julabo F25-HE refrigerated heating circulator. The initial hydration state of the sample (i.e., prehydration and predrying before loading into the sample chamber) is specified per (set of) experiment(s). Before each (set of) experiment(s), the sample chamber was evacuated at ∼20 °C for 30 min. During an experiment, the pressure in the sample chamber was increased stepwise with intermediate equilibration times of ∼10 min from vacuum (ultimate vacuum of the pump: 0.007 bar) up to ∼10 bar, while continuously monitoring the pressure (two Gems 3100 digital pressure transducers; 0–25 bar; accuracy: 0.25% of full-scale) and temperature in the sample chamber and reservoir. The adsorbed quantity of CO_2_ was calculated using the van der Waals equation of state to account for the nonideality of the gas.

## 3 Results and Discussion

### 3.1 Thermogravimetric Analysis

**Figure 2:**
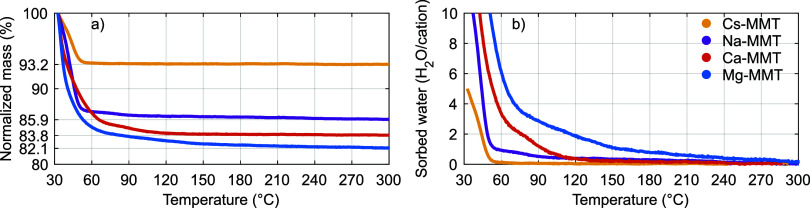
Thermogravimetric analysis (heating rate: 1 °C min^–1^) of H_2_O desorption from the MMT samples (prehumidified at ∼94% RH), with (a) normalized mass and (b) sorbed H_2_O per interlayer cation (on the basis of the reported structure of MMT^41^), as a function of temperature.

Thermogravimetric H_2_O desorption experiments of the MMT samples (prehumidified at ∼94% RH; heating rate: 1 °C min^–1^) reveal that the total H_2_O (de)sorption (Figure 2a) and the typical release temperature of the final H_2_O (Figure 2b; on the basis of the reported structure of MMT^41^) increase with increasing absolute hydration energy of the cation (Mg^2+^ > Ca^2+^ > Na^+^ ≫ Cs^+^). The total desorbed H_2_O is 12.1, 10.7, 9.1, and 4.0 mmol g^–1^, respectively. This is in line with the reported ability of Mg-, Ca-, and Na-MMT and inability of Cs-MMT to adsorb two planes of H_2_O in their interlayer galleries.^12,13,15,16,38^ Based on the domains with the different slopes in Figure 2b, we can distinguish several distinct dehydration steps. The initial desorption of weakly sorbed H_2_O is rapid and completed around 70, 60, 55, and 55 °C for Mg-, Ca-, Na-, and Cs-MMT, respectively. The remaining number of sorbed H_2_O molecules at the respective temperatures is around 4.1, 3.5, 1.0, and 0.2H_2_O per interlayer cation, respectively. This suggests that the interlayer cations in Mg- and Ca-MMT retain their first hydration shell (∼4H_2_O per interlayer cation^46,47^) under mild conditions due to their higher hydration energy, while the interlayer cations in Na- and Cs-MMT cannot. For Mg-, Ca-, and Na-MMT, the rapid initial desorption is followed by two domains of a more gradual release. Ca- and Na-MMT retain ∼0.5H_2_O per interlayer cation at 110 °C and Mg-MMT retains ∼1H_2_O per interlayer cation at 150 °C. These final H_2_O molecules are released gradually during the remainder of the experiment. Our observations are in qualitative agreement with earlier reports,^12,13,24,48−51^ but shifted toward lower temperatures likely due to the rather gentle temperature ramp used here.

### 3.2 H_2_O Ad- and Desorption

**Figure 3:**
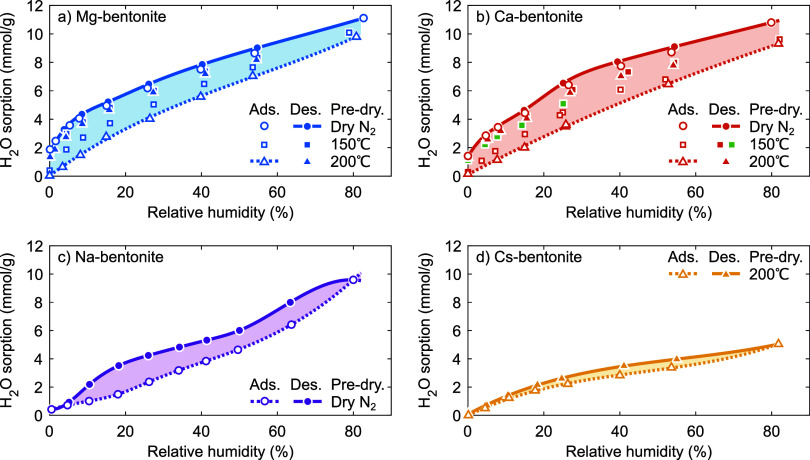
H_2_O sorption as a function of relative humidity for (a) Mg-bentonite, (b) Ca-bentonite, (c) Na-bentonite, and (d) Cs-bentonite. Open symbols and dashed lines indicate increasing relative humidity (i.e., the H_2_O adsorption branch), closed symbols and solid lines indicate decreasing relative humidity (i.e., the H_2_O desorption branch). In (b), the desorption branch in green corresponds to Ca-bentonite rehydrated at ∼40% RH, whereas all other samples were rehydrated at ∼80% RH.

Figure 3 shows the H_2_O sorption on the different bentonites under increasing (open symbols) and subsequently decreasing (closed symbols) RH conditions. The bentonites were predried under three different conditions: (i) under dry N_2_ purge at room temperature (circles), and at (ii) 150 °C (squares) or (iii) 200 °C (triangles) in an oven without gas purge. These elevated predrying temperatures cannot be compared directly to the thermogravimetric H_2_O desorption experiments in Figure 2 due to the absence of the dry N_2_ purge here. Generally, the H_2_O sorption increases with increasing RH and absolute hydration energy of the cation (Mg^2+^ ≈ Ca^2+^ > Na^+^ > Cs^+^; in agreement with the thermogravimetric H_2_O desorption experiments in Figure 2). The H_2_O sorption isotherms also depend on the predrying condition and the hydration history of the sample; all H_2_O sorption isotherms are bounded by (i) the H_2_O desorption branch of the samples predried at the lowest temperature (upper bound; solid lines), and (ii) the H_2_O adsorption branch of the samples predried at the highest temperature (lower bound; dotted lines). We believe that the intermediate states that are indicated by the shaded areas are accessible via alternative (de)hydration pathways (e.g., refs (52–54)).

Smectites can show crystalline swelling from the dehydrated or collapsed state (denoted as 0W) to expanded states with the number *n* of interlayer H_2_O planes denoted as *n*W states (i.e., 1W, 2W, etc.).^55,56^ The shapes of the sorption isotherms can be used to monitor this crystalline swelling, as swelling transitions and homogeneous hydration states show as steps and plateaus, respectively. These steps and plateaus can be obscured by (i) heterogeneity (i.e., the coexistence of multiple hydration states within the same sample or interlayer gallery due to structural or compositional variations), and by (ii) adsorption on external surfaces and (iii) condensation in meso- or macropores at high RH (∼80%), e.g., refs (12,13,28,38,56–62). The shapes of the H_2_O sorption isotherms that we measured are in good agreement with earlier reports.^12−14,28,38,39,52−54,57,63−66^ However, the maximum H_2_O sorption (at 80% RH) is somewhat lower than in most of these earlier reports for the respective MMTs, most likely due to the reduced MMT fraction (∼72%) of the bentonites used in this study.

The H_2_O sorption isotherms of Mg- and Ca-bentonite are highly similar and nearly concave (Figure 3a,b). Previous studies on Mg- and Ca-MMT suggest that these bentonites are in a predominant 2W state at 80% RH.^13,15,16,38,39,48,56,57,63,64,67−69^ For Ca-bentonite, the H_2_O desorption branches (closed symbols in Figure 3b) and H_2_O adsorption branch after predrying under dry N_2_ purge (open circles in Figure 3b) show a step at ∼20% RH. This step was reported earlier and coincides with the 2W–1W transition (or vice versa in the adsorption branch).^13,38,39,63,69^ For Mg-bentonite, this transition occurs at lower RH (e.g., refs (13 and 16)). The H_2_O adsorption branches show no steps (open symbols; except Ca-bentonite after predrying under dry N_2_, open circles in Figure 3b), which suggests a higher degree of heterogeneity during adsorption than during desorption. Indeed, earlier studies on Mg- and Ca-smectites reported the (nearly) simultaneous appearance of 1W and 2W states along the H_2_O adsorption branch.^13,38,69^

The H_2_O sorption isotherms of Na-bentonite (Figure 3c) show a maximum (at 80% RH) of 9.6 mmol g^–1^; nearly the maximum H_2_O sorption on the (2W) Mg- and Ca-bentonites. This observation is in line with earlier reports on Na-smectites that found a predominant 2W state in this RH domain.^12,15,38,39,52,54,56,57,63−65,67−70^ The H_2_O desorption branch shows steps at ∼60% RH and at ∼15% RH (closed circles). These steps were reported earlier and coincide with the 2W–1W and 1W–0W transitions, respectively.^12,28,38,52,54,63,65,69^ The H_2_O adsorption branch shows no steps (open circles), again likely due to the previously reported high(er) degree of heterogeneity during adsorption (than during desorption).^12,60,65,69^ Earlier studies on Na-smectite reported the progressive appearance of 1W and 2W states along the H_2_O adsorption branch at ≳20% RH.^12,15,38,39,54,57,65,67−69^

The H_2_O sorption isotherms of Cs-bentonite (Figure 3d) have a nearly concave shape, however, with a lower maximum (at 80% RH) than the other bentonites. This lower maximum is consistent with Figure 2 and the previously reported inability of Cs-smectites to adsorb two planes of H_2_O in their interlayer galleries.^12,16,38,39,71,72^ Due to the large size of Cs^+^, upon increasing the RH, H_2_O progressively fills the interlayer galleries without expanding them much further (i.e., from ∼11 to ∼12 Å).^12,14,16,21,26,38,39^ Above 40% RH, the sample is likely dominated by H_2_O-filled or nearly filled 1W states (e.g., refs (38 and 39)).

Figure 2 demonstrated that the higher the hydration energy of the exchanged cation, the higher the ability of the respective MMT or bentonite to retain H_2_O at somewhat elevated temperatures, most likely in the first hydration shell of these cations. To test if the samples also retain H_2_O under dry N_2_ purge, we (post)dried them at 200 °C after completing the desorption branches. These experiments revealed that Mg- and Ca-bentonite retained about 1.6 ± 0.3 and 1.3 ± 0.1 mmol H_2_O per g, respectively (5.5H_2_O/Mg^2+^ and 4.5H_2_O/Ca^2+^), whereas Na- and Cs-bentonite retained only ∼0.4 mmol H_2_O per g (0.7H_2_O/Na^+^) and essentially no H_2_O, respectively. Thus, analogous to Figure 2, these experiments suggest that Mg^2+^ and Ca^2+^ retain approximately their first hydration shell under dry N_2_ purge due to their higher hydration energy, whereas Na^+^ and Cs^+^ do not. This (in)ability to retain H_2_O is in agreement with earlier studies of smectite dehydration under a dry gas purge.^55,66−68,73^

Importantly, the H_2_O adsorption–desorption hysteresis depends on both the cation species and predrying conditions. H_2_O adsorption–desorption hysteresis has often been attributed to (free) energy barriers between hydration states.^71,72,74−76^ Therefore, it is generally oppressed when large cations (e.g., Cs^+^ and tetramethylammonium) open the interlayer galleries to reduce or remove swelling requirements toward H_2_O adsorption.^11,29,38,77^ For Cs-bentonite, we indeed find that the H_2_O adsorption–desorption hysteresis is limited over the complete range of RH (<0.6 mmol g^–1^; Figure 3d). For Mg- and Ca-bentonite, however, the H_2_O adsorption–desorption hysteresis increases with increasing predrying temperature, Figure 3a,b. This increase is mostly due to the lower H_2_O sorption along the adsorption branches after drying at elevated temperature (open symbols); the H_2_O desorption branches in turn demonstrate high reversibility after rehydration at sufficiently high RH (closed symbols; i.e., they are nearly independent of predrying conditions; cf. the sample rehydrated at ∼40% RH, green closed squares in Figure 3b). For the samples predried at 200 °C (triangles), the hysteresis is about 2.5 mmol g^–1^ at intermediate RH. For Na-bentonite predried under N_2_, it is similarly high and up to ∼2.0 mmol g^–1^, Figure 3c. Thus, these observations suggest that these well-dried and initially anhydrous bentonites are subject to free energy barriers along the H_2_O adsorption branches due to swelling requirements.^18,39,76,78^ In contrast, Mg- and Ca-bentonite predried under N_2_ (circles in Figure 3a-b) show remarkably limited H_2_O adsorption–desorption hysteresis over the complete range of RH, similar to Cs-bentonite and in agreement with earlier reports.^66,73^ This limited hysteresis suggests that interlayer H_2_O that is retained in the first hydration shell of the respective cations after the relatively mild drying under N_2_ (see above) opens the interlayer galleries to remove swelling requirements and facilitate H_2_O adsorption. This was also suggested in an earlier report^66^ and is analogous to the large Cs^+^ cations in Cs-bentonite.

### 3.3 CO_2_ Adsorption

**Figure 4:**
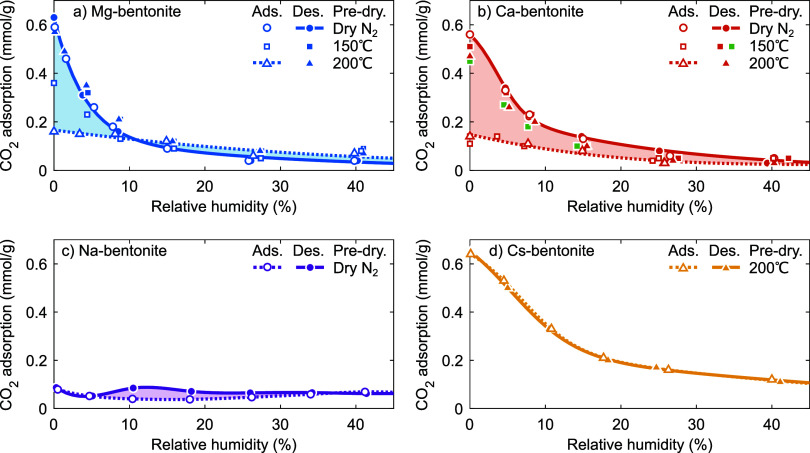
CO_2_ adsorption (at 1 bar, ∼20 °C) as a function of relative humidity for (a) Mg-bentonite, (b) Ca-bentonite, (c) Na-bentonite, and (d) Cs-bentonite. Open symbols and dashed lines indicate the CO_2_ adsorption measured after increasing the relative humidity (i.e., as determined along the H_2_O adsorption branch), closed symbols and solid lines indicate the CO_2_ adsorption measured after decreasing the relative humidity (i.e., as determined along the H_2_O desorption branch). In (b), the CO_2_ adsorption in green was determined along the H_2_O desorption branch of Ca-bentonite rehydrated at ∼40% RH, whereas all other samples were rehydrated at ∼80% RH.

Figure 4 shows the CO_2_ adsorption (at 1 bar, ∼20 °C) along the H_2_O adsorption and desorption branches in Figure 3. The CO_2_ adsorption on Mg-, Ca-, and Cs-bentonite generally decreases with increasing RH; however, for Mg- and Ca-bentonite it depends strongly on the predrying condition and/or the hydration history of the sample. Mg- and Ca-bentonite show the highest CO_2_ adsorption along the H_2_O desorption branches (closed symbols) and the H_2_O adsorption branch after predrying under N_2_ (open circles), with a maximum of ∼0.6 mmol g^–1^ under dry conditions that is similarly high as on Cs-bentonite. For these samples, the reduction of the CO_2_ adsorption with increasing RH is the more pronounced the higher the hydration energy of the cation. The CO_2_ adsorption along the H_2_O adsorption branches of Mg- and Ca-bentonite predried at 200 °C and on Na-bentonite (under all conditions) is much lower, with a maximum of ∼0.10–0.15 mmol g^–1^ under dry conditions. We believe that all states indicated by the shaded areas are accessible via alternative (de)hydration pathways, similar to H_2_O sorption.

**Figure 5:**
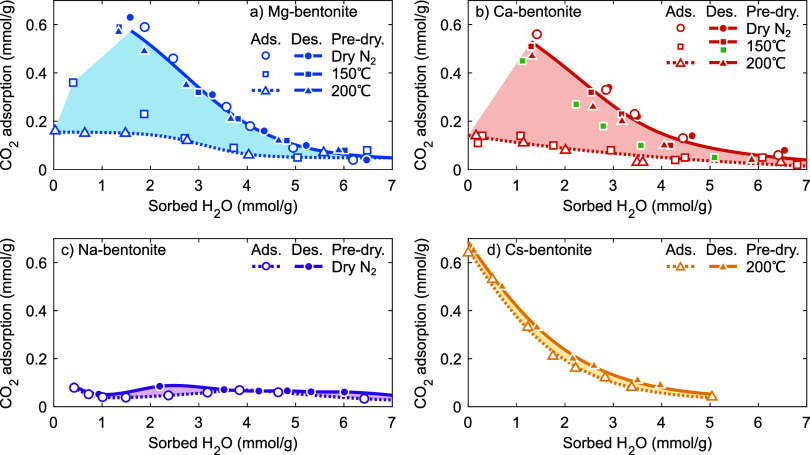
CO_2_ adsorption (at 1 bar, ∼20 °C) as a function of sorbed H_2_O for (a) Mg-bentonite, (b) Ca-bentonite, (c) Na-bentonite, and (d) Cs-bentonite. Open symbols and dashed lines indicate increasing relative humidity (i.e., as determined along the H_2_O adsorption branch), closed symbols and solid lines indicate decreasing relative humidity (i.e., as determined along the H_2_O desorption branch). In (b), the CO_2_ adsorption in green was determined along the H_2_O desorption branch of Ca-bentonite rehydrated at ∼40% RH, whereas all other samples were rehydrated at ∼80% RH.

To illustrate competitive and/or synergistic effects between the adsorption of H_2_O and CO_2_, Figure 5 shows the CO_2_ adsorption (as in Figure 4) as a function of the amount of sorbed H_2_O (as in Figure 3). For Mg- and Ca-bentonite, the CO_2_ adsorption again strongly depends on the predrying condition and/or the hydration history of the sample. However, for all H_2_O desorption branches (closed symbols) and the H_2_O adsorption branch after predrying under N_2_ (open circles), these bentonites now display nearly identical decrease of the CO_2_ adsorption with increasing H_2_O sorption (around −0.19 CO_2_ per H_2_O). This decrease is, however, accompanied by a small shift which could tentatively be the result of the somewhat larger bare cation size of Ca^2+^ (Table S1), its somewhat lesser ability to bind and organize H_2_O and/or CO_2_ molecules,^79,80^ and differences in their reference “dry” state (i.e., oven-dried at 200 °C, while some H_2_O is only released at higher temperatures, Figure 2). For Cs-bentonite, the decrease of the CO_2_ adsorption with increasing H_2_O sorption is also shifted and initially steeper (around −0.23 CO_2_ per H_2_O) than for Mg- and Ca-bentonite. We tentatively attribute these differences to a less-efficient packing of CO_2_ in the interlayer galleries of Cs-bentonite, possibly due to the presence of twice as many (large) Cs^+^ as Mg^2+^ or Ca^2+^ cations to compensate for the layer charge.^21,23,80,81^ Alternatively, this could be due to the lesser ability of Cs^+^ than Mg^2+^ and Ca^2+^ to bind and organize H_2_O and/or CO_2_ molecules.^79,80^

**Figure 6:**
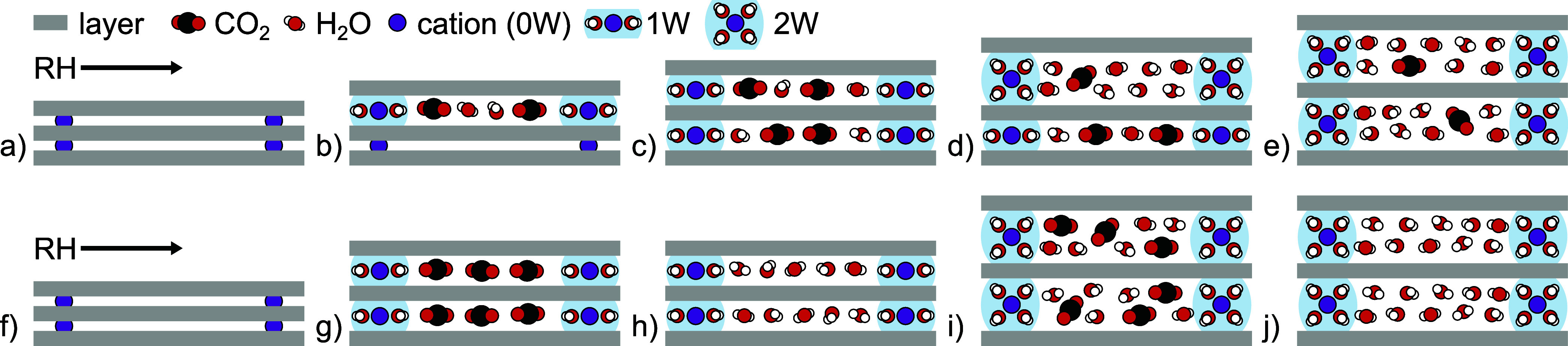
Sequential hydration paths for smectites in the presence of CO_2_ following the previously proposed (a–e) hydration-heterogeneity and (f–j) space-filling models. (Schematic and not to scale.) In (a–e), with increasing RH, the hydration states follow the order (a) 0W, (b) mixed 0W/1W, (c) 1W, (d) mixed 1W/2W, and (e) 2W. Herein, the CO_2_ adsorption is not permitted in the 0W state, at a maximum in the 1W state, and low in the 2W state. In (f–j), with increasing RH, the hydration state transitions homogeneously from (f) the 0W collapsed state, to (g) a 1W state with hydrated cations only that permits high CO_2_ adsorption, to (h) a filled 1W state with little or no CO_2_ adsorbed, to (i) a relatively empty 2W state with high CO_2_ adsorption, to (j) a filled 2W state with little or no CO_2_ adsorbed.

As discussed in the Introduction, the adsorption of CO_2_ in the interlayer galleries of smectites can be facilitated when these are sufficiently opened either by some cosorbed H_2_O,^3,5,6,9,17−19,21,24−26,40^ or by sufficiently large cations (in the absence of adsorbed H_2_O; e.g., Cs^+^).^1,21−23,25−27,30,82^ Two conceptual models were proposed earlier for CO_2_ adsorption in interlayer galleries that are opened by cosorbed H_2_O.^5,9^ The hydration-heterogeneity model (Figure 6a–e) assumes that a sample contains a linear combination of interlayer galleries with integral hydration states (i.e., ∼*n*W) that each contain fixed but different amounts of adsorbed CO_2_ under the given temperature and pressure conditions. The highest CO_2_ adsorption is often assumed for the 1W state, and less and none for the 2W and 0W states, respectively.^9,21,83,84^ The proportions of *n*W states shift with RH and could thereby explain a variation of CO_2_ adsorption with RH. The space-filling model (Figure 6f–j) instead assumes that a sample is homogeneously hydrated and posits that the swelling and adsorption of H_2_O occur in two steps: (i) hydration of the interlayer cations and swelling of the interlayer gallery without completely filling it, and then (ii) filling the remainder of the interlayer gallery at higher RH without further swelling. The interlayer voids created after step (i) then allow for CO_2_ adsorption without significant further expansion of the smectite (similar to step (ii) for H_2_O). Both conceptual models were, however, discussed in the context of scCO_2_ (see Section 3.4) and disregard that the adsorption of H_2_O can occur in two steps with CO_2_ adsorbed in the interlayer voids, but heterogeneously between interlayer galleries. The mechanism may furthermore depend on the specific interlayer cation species (e.g., ref (71)) and may differ between the H_2_O adsorption and desorption branches and the 0W–1W and 1W–2W transitions. Therefore, the mechanism(s) may not be unifiable in a single conceptual model.

We first consider CO_2_ adsorption under anhydrous conditions. Previous studies showed that the interlayer galleries of Cs-smectites are then readily accessible to CO_2_ due to the large size of Cs^+^,^1,22,23,25−27,82^ whereas the interlayer galleries of Mg-, Ca-, and Na-smectites are then inaccessible to CO_2_.^1,6,9,17−27^ Our observations on the anhydrous bentonites at 0% RH are fully consistent with these previous studies; specifically the high CO_2_ adsorption of ∼0.6 mmol g^–1^ on Cs-bentonite (Figure 4d) and the low CO_2_ adsorption of ≲0.15 mmol g^–1^ on Mg-bentonite predried at 200 °C (open triangles in Figure 4a), Ca-bentonite predried at 150 and 200 °C (open squares and open triangles in Figure 4b, respectively), and Na-bentonite predried under N_2_ (Figure 4c).

We now consider the H_2_O adsorption branches, starting from the initially anhydrous 0W state described above and upon increasing the RH. For Cs-bentonite (open triangles in Figure 4d), H_2_O progressively fills the interlayer galleries at higher RH, effectively outcompeting CO_2_. The CO_2_ adsorption reduces to ≲0.1 mmol g^–1^ above ∼40% RH, where the sample is likely dominated by H_2_O-filled or nearly filled 1W states. These states, thus, do not allow for high CO_2_ adsorption. For Na-bentonite (open circles in Figure 4c), we find that the (already low) CO_2_ adsorption initially decreases with increasing RH. At low RH, H_2_O was previously shown to adsorb predominantly on the external surfaces of Na-smectites.^12,28,38,39,53,54,57−60,66,85^ Therefore, this decrease is likely due to the competitive adsorption with H_2_O on external surfaces. The CO_2_ adsorption then passes through a low and broad maximum around 30–55% RH, i.e., corresponding to the appearance of 1W and possibly some 2W states. Finally, the CO_2_ adsorption decreases again when the Na-bentonite evolves toward a predominant 2W state at the highest RH. For Mg- and Ca-bentonite, the swelling from the 0W state to the (eventually) 2W state occurs at much lower RH than for Na-bentonite (e.g., refs (13–16,38,39,57, and 69)); the net effect is a gradual decrease of the CO_2_ adsorption with increasing RH (open triangles in Figure 4a, open squares and triangles in Figure 4b).

However, CO_2_ adsorption along the H_2_O adsorption branch of initially anhydrous Mg-, Ca-, and Na-bentonite is much lower than on anhydrous Cs-bentonite (and in the H_2_O desorption branches of Mg- and Ca-bentonite to be discussed below). This suggests that at any given RH, only a small fraction of the interlayer space within a sample can be occupied by CO_2_. This could be compatible with the conceptual hydration-heterogeneity model (Figure 6a–e) if the *n*W hydration states as they are assumed in that model permit relatively little CO_2_ adsorption under the low pressure conditions studied in this work. Indeed, we find that Cs-bentonite adsorbs little CO_2_ when it is predominantly in a 1W state at high RH. There is no reason to assume that the 1W states (i.e., as they are assumed in the hydration-heterogeneity model) of Mg-, Ca-, and Na-bentonite adsorb significantly more CO_2_. Similarly, we find that Mg-, Ca-, and Na-bentonite adsorb little CO_2_ when they are predominantly in a 2W state at high RH. These observations imply that any high CO_2_ adsorption on Mg-, Ca-, and Na-bentonite, if observed at all, should occur in the voids between hydrated cations around the *n*W transitions instead (Figure 6g,i), as in the space-filling model. That we do not observe a high CO_2_ adsorption along the H_2_O adsorption branches of these bentonites could still be compatible with a space-filling-like model, i.e., with the CO_2_ adsorption only permitted in the voids between hydrated cations, if (i) the H_2_O adsorption does not occur in two steps and only the states that are largely filled by competitively adsorbed H_2_O and hence do not contain such voids are probed along the H_2_O adsorption branch, and/or if (ii) the H_2_O adsorption occurs highly heterogeneously, such that at any given RH only a minor fraction of all interlayer galleries feature such voids with the remainder of the interlayer galleries either collapsed or filled by H_2_O. In support of item (i), the molecular simulations by Brochard^76^ demonstrated that the 1W state of Na-MMT at a RH close to the experimentally observed 0W–1W transition (∼20–30% RH)^12,15,38,39,54,57,64,65,67,68^ is relatively filled as it contains ≳75% of the H_2_O capacity of that state. Consequently, Na-smectites may transition upon increasing the RH from the collapsed 0W state directly to this relatively filled 1W state without passing through a (CO_2_-accessible) partially filled 1W state. Equivalent arguments apply to the 1W–2W transition and possibly to Mg- and Ca-smectites. Similarly, the molecular simulations by Rao and Leng^19^ showed that H_2_O molecules readily fill the interlayer gallery of Na-MMT after its expansion. The molecular simulations by Young and Smith^71^ furthermore demonstrated that the 1W–2W transition for smectites with monovalent interlayer cations does not occur in two steps. However, their simulations do show that a two-step 1W–2W transition is possible for Sr-MMT. We could expect similar behavior for Mg- and Ca-smectites and possibly the 0W–1W transition for divalent cations. However, in support of item (ii), previous studies indicated that the H_2_O adsorption on Mg-, Ca-, and/or Na-smectites occurs heterogeneously with the coexistence of 0W, 1W, and/or 2W states at intermediate RH.^12,13,38,54,60,67−69^ This heterogeneity is also suggested by the absence of steps along the H_2_O adsorption branches that we measured (Figure 3a–c).

We now consider the H_2_O desorption branches, starting from the fully hydrated 2W (Mg-, Ca-, and Na-bentonite) or 1W (Cs-bentonite) state at ∼80% RH and upon decreasing the RH (closed symbols). For Cs-bentonite (Figure 4d), we find that the CO_2_ adsorption is indifferent between the H_2_O adsorption and desorption branches. This observation is in accordance with its nearly absent H_2_O adsorption–desorption hysteresis. For Na-bentonite (Figure 4c), we find that the CO_2_ adsorption first increases around the 2W–1W transition (∼60% RH; Figure 3c), then saturates into a plateau value around the predominant 1W state (∼50–18% RH), and subsequently peaks around the 1W–0W transition (∼15–9% RH). Finally, the CO_2_ adsorption increases at the lowest RH, likely due to the now reduced competitive adsorption on external surfaces. The higher degree of homogeneity in the H_2_O desorption branch than in the H_2_O adsorption branch (Figure 3c) likely resulted in the somewhat increased CO_2_ adsorption around the 2W–1W and 1W–0W transitions. This observation is mostly compatible with a space-filling-like model. Nevertheless, the CO_2_ adsorption on Na-bentonite never approached that on Cs-bentonite. Indeed, previously reported (differential) thermograms of H_2_O desorption from Na-MMT often suggest that (most of the) H_2_O from the first hydration shell of the cation is removed nearly simultaneous with H_2_O outside of this first hydration shell (i.e., H_2_O desorption does not occur in two steps, Figure 2).^24,48−51,86^

For Mg- and Ca-bentonite (Figure 4a,b), we find that the CO_2_ adsorption along the H_2_O desorption branches is nearly independent of the predrying condition. This observation is in accordance with the high reversibility of the H_2_O desorption branches (Figure 3a,b). Similar to Na-bentonite, it is low until these materials are likely dominated by relatively filled 1W states (at 13–7% RH for Mg-bentonite and 16–11% RH for Ca-bentonite; Figure 3a,b and, e.g., refs (13,38, and 39)). Indeed, Ferrage et al.^46,47^ suggested for the 2W–1W transition of Ca-MMT a local collapse that occurs in the presence of H_2_O not coordinated to the cations and thereby the coexistence of relatively filled 1W and 2W domains within the same interlayer gallery, as well as heterogeneity between different interlayer galleries. Consequently, during the 2W–1W transition, the Ca-bentonite mostly lacks the voids that are required for CO_2_ adsorption and this conclusion likely extends to Mg-bentonite. However, upon further decreasing the RH, the CO_2_ adsorption on Mg- and Ca-bentonite increases and even approaches the maximal CO_2_ adsorption on Cs-bentonite at ∼0% RH. In contrast to Na-MMT, previously reported (differential) thermograms of H_2_O desorption from Mg- and Ca-MMT often suggest that H_2_O outside the hydration shell of the cations is removed first, while H_2_O in the hydration shell of the cations is retained in the sample until higher temperatures (i.e., dehydration does occur in two steps, Figure 2).^24,46−51,86−88^ Ferrage et al.^46,47^ demonstrated that further dehydration (in their case for Ca-MMT and at higher temperature) then involves the successive removal of one H_2_O molecule from the hydration shell of each interlayer cation, that is likely accompanied by the rearrangement of the remaining H_2_O molecules. This rearrangement furthermore increases (slightly) the energy requirement to remove a subsequent H_2_O molecule,^51^ until the first hydration shell of around 4H_2_O is retained and strongly bound to each cation in a geometry resembling ‘a very flat tetrahedron’ (Ferrage et al.^46^ and refs therein). According to Ferrage et al.,^46,47^ the 1W–0W transition starts only after the limit of ∼4H_2_O/M^2+^ is reached with likely first the collapse of the interlayer around a cation and then the propagation of this collapse to adjacent cations. However, this 1W–0W transition requires a high(er) activation energy as compared to the desorption of H_2_O before the limit of ∼4H_2_O/M^2+^ is reached.^47,51^ Likely as a consequence of this high activation energy, the driest state obtained here under dry N_2_ purge for Mg- and Ca-bentonite still contained 1.6 mmol g^–1^ and 1.3 mmol g^–1^ sorbed H_2_O, respectively (5.5H_2_O/Mg^2+^ and 4.5H_2_O/Ca^2+^; much more than the 0.7H_2_O/Na^+^ for Na-bentonite; Figure 3a–c). Ferrage et al.^46^ furthermore found a ∼90% abundance of the 1W state at these H_2_O contents, i.e., the sample is hydrated relatively homogeneous between and within interlayer galleries. The voids between the hydrated cations (that are again analogous to the large Cs^+^ cations in Cs-bentonite) then provide the high CO_2_ adsorption capacity of ∼0.6 mmol g^–1^ of these samples, as was also suggested in earlier reports.^24,25^

When the RH is increased again from this state, i.e., along the H_2_O adsorption branch of Mg- and Ca-bentonite predried under N_2_ (open circles in Figure 4a,b) and similarly for Cs-bentonite (Figure 4d), the CO_2_ adsorption equals that along the H_2_O desorption branches. This indifference is in accordance with the limited H_2_O adsorption–desorption hysteresis of these samples (Figure 3a,b,d). Note also that Mg-bentonite predried at 150 °C (open squares in Figure 4a) shows behavior intermediate between that predried at 200 °C and that predried under N_2_, with a maximum CO_2_ adsorption of ∼0.36 mmol g^–1^ under dry conditions. This suggests that the predrying of Mg-bentonite at 150 °C only removes (part of the) H_2_O from the first hydration shell of (part of) the cations.

### 3.4 Comparison with High-Pressure CO_2_ Adsorption

The (co)adsorption of H_2_O and gaseous, low-pressure CO_2_ as in the present Article is mostly compatible with a space-filling-like model. In contrast, previous studies considered the (co)adsorption of H_2_O and high-(partial)pressure CO_2_ (e.g., supercritical [sc]CO_2_) to be more compatible with the hydration-heterogeneity model.^5,9,19,21^ Two pressure effects can explain this difference. First, higher CO_2_ pressures promote the swelling of an initially collapsed smectite with small interlayer cations^7,18,26^ by the mutually synergistic adsorption of CO_2_ and H_2_O. Second, higher CO_2_ pressures increase the CO_2_ adsorption and/or lower the H_2_O sorption at a given RH for an already expanded (either by hydrated or by large cations) smectite.^18,26,89,90^ Consequently, the adsorption of variably wet scCO_2_ on initially collapsed Na-MMT under increasing RH (or, more specifically, the percent H_2_O saturation in the scCO_2_) can be as high as ∼0.8 mmol g^–1^^26^ (yet, this is significantly less than on Cs-MMT under equivalent conditions). This adsorption is reduced to nearly none when the CO_2_ is instead adsorbed from a variably wet scCH_4_–CO_2_ mixture containing 3% or 25% CO_2_, in accordance with our observations. Similarly, the RH-domain in which significant CO_2_ sorbs on Cs-^21,26^ and on Ca-smectites^9,21^ is, compared to this work, extended to higher RH when the CO_2_ is instead adsorbed from variably wet scCO_2_. Contrary to the active role of high-pressure (sc)CO_2_ with respect to the swelling of collapsed smectites and the displacement of H_2_O from expanded smectites, we thus see a more passive (space-filling) role for low-pressure (gaseous) CO_2_.

### 3.5 Stability of Adsorbed H_2_O

The preceding sections demonstrated that the hydration state of Mg- and Ca-bentonite with hydrated interlayer cations and without competitively adsorbed H_2_O outside the first hydration shell of the cations is conducive to high CO_2_ adsorption. We now consider the stability of this hydration state under some conditions that may result in the removal of the remaining synergistically adsorbed H_2_O from the cation hydration shell and thus the loss of the CO_2_ adsorption capacity. This assessment is important for the purpose of using the bentonites as (cyclic) CO_2_ adsorbents, as the removal of the remaining H_2_O would require the subsequent regeneration at sufficiently high RH (Figure 4a,b) into a state with hydrated cations to fully restore their CO_2_ adsorption capacity. We exclude Na-bentonite from this analysis for the low CO_2_ adsorption thereon under the relevant conditions.

**Figure 7:**
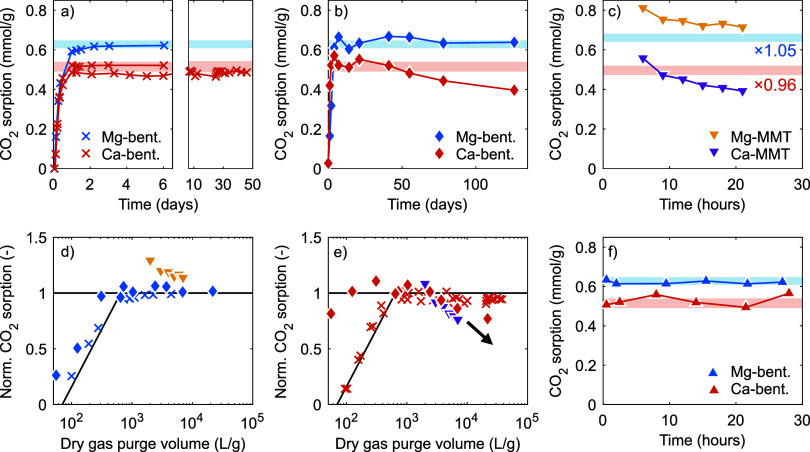
(a,b) CO_2_ adsorption (at 1 bar, ∼20 °C) as a function of dry gas purging time for Mg- and Ca-bentonite, with (a) method (i) and (b) method (ii). (c) CO_2_ adsorption (at 0.8 bar, 30 °C) as a function of dry gas purging time for Mg- and Ca-MMT, method (iii). (d,e) Normalized (by the plateau value, see below) CO_2_ adsorption as a function of dry gas purge volume per g sample (data from and legend as in (a–c)), on Mg- and Ca-bentonite and -MMT, respectively. (f) CO_2_ adsorption (at 1 bar, 20 °C) as a function of evacuation time (at ∼0.007 bar), method (iv). The light-shaded bands in (a–c,f) indicate the (average) plateau value from methods (i–ii,iv) and are corrected for sample purity and experimental conditions in (c). (We refer to the main text for a detailed description of the different methods).

First, we assess the stability of the CO_2_ adsorption capacity under extended dry gas purge conditions. To this end, Figure 7a, method (i), presents the CO_2_ adsorption (at 1 bar, ∼20 °C) on Mg- and Ca-bentonite (3.5–3.9 g, prehumidified at ∼80% RH) as a function of dry N_2_ purging time, as determined using the balance setup (Section 2.2). Similarly, Figure 7b, method (ii), presents the CO_2_ adsorption (at 1 bar, 20 °C) on Mg- and Ca-bentonite (prehumidified at ∼90% RH) as a function of ex situ dry N_2_ purging time. In short, for method (ii), multiple samples of both Mg- and Ca-bentonite, ∼6.5 g each, were placed in a larger container that was continuously purged with dry N_2_ (∼2.7 L min^–1^). A sample was taken from this container regularly for analysis in the Sieverts apparatus (Section 2.4). The CO_2_ adsorption at 1 bar is interpolated from a dual-site Langmuir fit to an adsorption isotherm (to be discussed in Section 3.6 hereafter). Further details about the ex situ drying and method (ii) are provided in the Supporting Information.

The CO_2_ adsorption on these bentonites, methods (i–ii), first increases within around 2 days to a plateau value of around 0.62 mmol g^–1^ and 0.51 mmol g^–1^ for Mg- and Ca-bentonite, respectively (indicated by the light-shaded bands in Figure 7a–c,f). This plateau value is close to the maximum adsorption found in Figure 4a,b, as expected. We attribute the initial increase to the removal of competitively adsorbed H_2_O outside the first hydration shell of the interlayer cations. At longer N_2_ purge times, the CO_2_ adsorption on Mg-bentonite (methods (i-ii)) and on Ca-bentonite (method (i) only) remains at this plateau value. This indicates that during the remainder of the experiment no H_2_O is removed from the first hydration shell of the cations. The CO_2_ adsorption on Ca-bentonite dried ex situ (method (ii)), however, does decrease gradually after several weeks under N_2_ purge to 77% of the plateau value after 126 days. The interlayer space in this sample that was available to CO_2_ around the plateau thus appears to be reduced by the removal of some H_2_O from the first hydration shell of (some of) the cations.

Figure 7c, method (iii), presents the CO_2_ adsorption (at 0.8 bar, 30 °C) on Mg- and Ca-MMT (∼18 mg, prehumidified above liquid H_2_O) as a function of dry gas purging time, as determined using the thermogravimetric analyzer (Figure S2; Section 2.3). The CO_2_ adsorption on Mg-MMT decreases gradually from 0.81 mmol g^–1^ after 6 h to 0.71 mmol g^–1^ after 21 h and remains above the plateau value for Mg-bentonite (i.e., after correcting for the lower sample purity and the different experimental conditions of the latter). In contrast, the CO_2_ adsorption on Ca-MMT after 6 h (0.56 mmol g^–1^) is near the corrected plateau value for Ca-bentonite and decreases (faster than Mg-MMT) to 0.39 mmol g^–1^ after 21 h, similar to Ca-bentonite using method (ii).

Remarkably, the decrease of the CO_2_ adsorption on Ca-MMT in the thermogravimetric setup to ∼76% of the (corrected) plateau value occurred within a day (method (iii), Figure 7c), whereas a similar decrease to ∼77% of the plateau value on Ca-bentonite dried ex situ only occurred after 126 days (method (ii), Figure 7b). As the ratio between the gas flow rate and sample mass can vary by up to 2 orders of magnitude between the different methods (∼32 L h^–1^ g^–1^ for method (i); ∼2–12 L h^–1^ g^–1^ for method (ii); ∼333 L h^–1^ g^–1^ for method (iii)), this suggests that rather the dry gas purge volume (per unit sample mass) determines the removal of H_2_O, Figure 7d,e. From Figure 7e, we can very conservatively estimate that at the very least 10^4^ L of dry gas is required to remove 1 mmol of H_2_O (i.e., the estimated remaining H_2_O content at the plateau value, see Figure 5) from the first hydration shell of the interlayer cations in 1 g of Ca-bentonite or -MMT at 20–30 °C to collapse the interlayer galleries and reduce the CO_2_-accessible interlayer space. This corresponds, on average, to a H_2_O (partial) pressure of ∼0.24 Pa (or ∼0.01% RH at 20 °C). Ferrage et al.^56^ noted that Mg- and Ca-MMT can retain a predominant 1W state in a ∼10^–4^ Pa vacuum. Their observations suggest that considerably more dry gas than our estimate is required. Similarly, Ziemiański et al.^25^ showed that Mg-MMT can retain a CO_2_ adsorption capacity similar to Cs-MMT after degassing for 20 h at 60 °C in a ∼10^–3^ Pa vacuum (even though Ca-MMT cannot). These earlier observations and the proposed dehydration mechanism furthermore provide a reasonable explanation for the nondecreasing CO_2_ adsorption on Ca-bentonite studied using method (i), Figure 7a; trace amounts of H_2_O (despite our best efforts to remove sources thereof) likely provided a sufficiently high H_2_O pressure to stabilize the H_2_O in the first hydration shell of the cations in this sample.

Second, we assess the stability of the CO_2_ adsorption capacity under extended evacuation conditions. To this end, the sample from Figure 7b that was dried ex situ under N_2_ purge for three (Mg-bentonite) or two (Ca-bentonite) weeks was exposed to the vacuum for an extended time, method (iv). The CO_2_ adsorption (at 1 bar, 20 °C) was determined regularly (as in method (ii)) and is presented as a function of the evacuation time in Figure 7f. The CO_2_ adsorption starts from the plateau values (as expected) and does not decrease during the prolonged evacuation at a vacuum pressure of ∼0.007 bar. Indeed, this vacuum pressure is orders of magnitude higher than required to remove H_2_O from the first hydration shell of the cations as per our conservative estimate.

Third, in Figure S3, method (v), we assess the stability of the CO_2_ adsorption capacity similar to method (iii), but with intermediate drying at elevated temperatures. After 6–9 h under dry gas purge at 30 °C, the CO_2_ adsorption on Mg- and Ca-MMT is nearly equal to method (iii), as expected. After 12–18 h under dry gas purge, including 2–3 h at 90 °C, the CO_2_ adsorption on Mg- and Ca-MMT is reduced to 0.54–0.67 mmol g^–1^ and 0.13 mmol g^–1^, respectively. After almost 19–28 h, including 2–3 h at 150 °C, it is reduced further to only 0.16–0.29 mmol g^–1^ and 0.12 mmol g^–1^, respectively (i.e., similar to the anhydrous materials, Figure 4a,b). These results and Figures 2 and 5 suggest the progressive collapse of the interlayer galleries into a state inaccessible to CO_2_ upon drying at elevated temperatures due to the removal of H_2_O from the first hydration shell of the cations. We further note that a fraction of the CO_2_ is rather slowly adsorbed on and desorbed from (if at all at 30 °C) the MMTs that were dehydrated to <4H_2_O/M^2+^, e.g., Mg-MMT after drying at 90 °C (Figure S3b). While we can only speculate, this could possibly be due to (i) lower CO_2_ diffusion coefficients in interlayer galleries that contain less H_2_O,^10,18−20^ (ii) CO_2_ molecules that interact closely with cations that have an incomplete hydration shell (i.e., substituting for missing H_2_O),^9,91,92^ and/or (iii) the formation of more-tightly adsorbed species such as bicarbonate from cation-coordinating CO_2_ molecules.^92^ The direct coordination of the (divalent) cations by CO_2_ that is required for items (ii–iii) was, however, not observed experimentally^9,21^ nor in grand canonical simulations.^20,84^ Alternatively, this may be due an initial nonequilibrium distribution of H_2_O within and between the interlayer galleries that is redistributed during (and possibly facilitated by) the CO_2_ adsorption. This redistribution may foster cation-CO_2_ interactions as in items (ii–iii) and/or may trap CO_2_ in some domains of the interlayer galleries, while other domains are gradually opened up by the redistributed H_2_O.

Thus, the collapse of Mg- and Ca-bentonite into a state inaccessible to CO_2_ requires drying at elevated temperatures or, in the case of Ca-bentonite, very large amounts of dry gas purge. This is in line with numerous experimental evidence for incomplete drying of in particular Mg- and Ca-smectites under various, relatively mild, conditions (e.g., mild heating, evacuation, and/or dry gas purging). This evidence includes, for example, noncollapsed interlayer galleries,^9,13,17,21,24,25,34,40,55,56,67,68,80−82,85,93−96^ H_2_O adsorption–desorption hysteresis that does not vanish at ∼0% RH (in the case of initially collapsed MMT)^13^ or is nearly absent (in the case of MMT that was dehydrated mildly in the first place),^66^ and IR measurements of H_2_O.^9,10,21,73,81,96^ The different (de)hydration pathways are compiled in Figure S4, that combines the results in Figures 5, 7a,c, and S3.

### 3.6 CO_2_ Adsorption Isotherms

**Figure 8:**
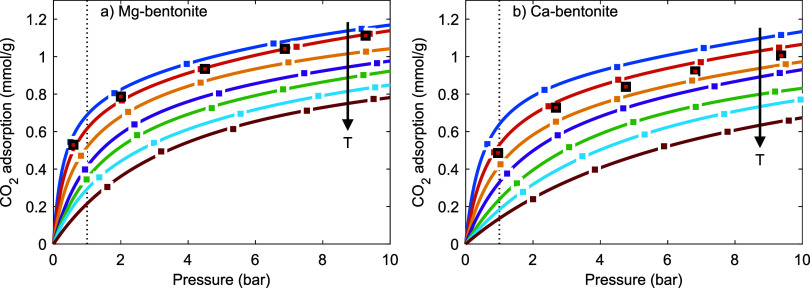
CO_2_ adsorption isotherms on (a) Mg-bentonite and (b) Ca-bentonite, fit with the (multisite) Langmuir equation (lines). Arrows indicate increasing temperature from 10 to 70 °C at 10 °C increments. The samples were predried ex situ under N_2_ purge for >2 weeks and evacuated for 1 h in between the isotherm measurements. Black squares indicate the second measurement at 20 °C.

For the purpose of using Mg- and Ca-bentonites as cyclic CO_2_ sorbents, we determined CO_2_ adsorption isotherms in the ranges *T* = 10–70 °C and *P* = 0–10 bar using the Sieverts apparatus (Section 2.4), Figure 8. (See ref (1) for CO_2_ adsorption isotherms on Na- and Cs-bentonite predried at 150 °C). The samples were predried ex situ at room temperature under dry N_2_ purge for several weeks. The isotherms were measured consecutively in the order 20, 10, 30...70 °C, and again 20 °C. In between the isotherm measurements, the samples were evacuated for 1 h at an intermediate temperature. The reproducibility of the measurement at 20 °C (black squares) indicates the ability of both materials to retain the H_2_O in the first hydration shell of the cations also during sample evacuation for >1 h at >60 °C to 0.007 bar. The isotherms are negatively curved in the low-pressure domain (see also Figures S5 and S6) and increase more gradually in the high-pressure domain ≳3 bar. The steepness in the low-pressure domain and the high-pressure capacity decrease with increasing temperature. We fit the adsorption isotherms with the Langmuir adsorption isotherm^97^
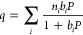
where *n*_*i*_ is the number of adsorption sites of type *i* per g sample and *b*_*i*_ is the equilibrium constant for sites of type *i*, with *i*∈{1,2} for interlayer galleries and external surfaces. The interpolated CO_2_ adsorption at 1 bar and 20 °C is 0.62 mmol g^–1^ and 0.53 mmol g^–1^ for Mg- and Ca-bentonite, respectively, and agrees well with the maximum CO_2_ adsorption in Figure 4a,b and the plateau value in Figure 7a,b,f, as expected. The maximum CO_2_ adsorption (at 10 °C and 10 bar) is ∼1.17 and ∼1.13 mmol g^–1^, respectively.

The isotherms are qualitatively similar to those of the CO_2_-accessible anhydrous Cs-, (mono)methylammonium- and tetramethylammonium-bentonite and -MMT that have a *d*-spacing of ∼11–14 Å (Table S1).^1,25,27^ Previous studies showed that the *d*-spacing of (sub-)1W Mg- and Ca-MMT dried under relatively mild conditions is ∼12 Å^9,17,21,24,25,40,46,80,94^ (more specifically, 12.0 Å for Ca-MMT with 4.5H_2_O/Ca^2+^).^46^ Consequently, the interlayer galleries of Mg- and Ca-bentonite should indeed be similarly accessible to CO_2_ as those of the anhydrous Cs-, (mono)methylammonium-, and tetramethylammonium-exchanged materials. The systematically slightly higher CO_2_ adsorption on Mg-bentonite than on Ca-bentonite can then be attributed the higher residual H_2_O content of Mg-bentonite than of Ca-bentonite (5.5H_2_O/Mg^2+^ and 4.5H_2_O/Ca^2+^) that likely results in a somewhat larger *d*-spacing for Mg-bentonite than for Ca-bentonite. The isotherms presented here are also in qualitative agreement with the isotherms presented by Grekov et al.^24^ on Mg-MMT (predried at 115 °C followed by outgassing in a ∼1 Pa vacuum) and by Schaef et al.^9,17^ on Ca-MMT (predried at 50 °C in a ∼10^–3^ Torr vacuum). They also agree qualitatively with the isotherms presented by Ziemiański et al.^25^ on Mg-MMT (predried at 60 °C in a ∼0.001 Pa vacuum), although the CO_2_ adsorption on their Ca-MMT predried under the same conditions (that are less mild than ours) was significantly lower. However, a quantitative comparison is hindered by differences in the studied temperature and pressure ranges or a limited resolution of the data in the overlapping ranges, and by differences in sample purity, sample origin, and predrying conditions.

## 4 Conclusions

Systematic experiments were performed to study the competitive and synergistic adsorption of CO_2_ and H_2_O in the interlayer galleries of smectites with the ultimate goal to use these materials as a sorbent to separate CO_2_ from mixed gas streams under near-ambient conditions. These experiments revealed a space-filling-like adsorption where H_2_O can be adsorbed synergistically and competitively with respect to CO_2_, depending on the (de)hydration history of the sample. This contrasts the mutually synergistic and competitive adsorption previously found at higher CO_2_ pressure. Specifically, high CO_2_ adsorption requires either sufficiently large (e.g., Cs^+^) or hydrated interlayer cations to provide a sufficiently wide interlayer gallery, and no competitively adsorbed H_2_O. The homogeneous hydration state with hydrated cations only was achieved by suitable pretreatment that involved dedicated hydration followed by mild drying (i.e., dry gas purge or a weak vacuum at near-ambient temperature) to remove the competitively adsorbed H_2_O only. Moreover, it was only found for smectites with strongly hydrated interlayer cations (e.g., Mg^2+^, Ca^2+^) that maintain a very stable first hydration shell under these mild drying conditions. Importantly, this hydration state was never encountered for weakly hydrated cations (e.g., Na^+^) because these cations lose their first hydration shell nearly simultaneous with the competitively adsorbed H_2_O, nor was it found for initially anhydrous smectites studied under increasing relative humidity conditions because of the specific heterogeneous and/or hysteretic nature of H_2_O adsorption. Thus, our experiments revealed that the CO_2_ adsorption depends not only on the previously shown interlayer cation species and thermodynamic variables as the temperature, CO_2_ pressure, and relative humidity, but also strongly on the specific (de)hydration history of the sample.

The high stability of the H_2_O in the first hydration shell of strongly hydrated cations enables the use of the respective (near-)natural, readily available and cheap bentonites for various CO_2_ adsorption and separation purposes. This requires (i) an overall low H_2_O content in the gas stream from which the CO_2_ is to be sorbed to prevent the competitive adsorption of H_2_O, and (ii) sorbent regeneration that is not performed at (strongly) elevated temperatures. Fortunately, for item (ii), the favorable shape of the CO_2_ adsorption isotherms that are similar to the more expensive Cs-bentonite^1^ suggest that these sorbent materials desorb CO_2_ and regenerate easily in a weak vacuum at room temperature. Moreover, earlier work also suggested lower capital and operational costs of CO_2_ separation compared to conventional sorbents when using Cs-bentonite. The current work suggests a further reduction of material costs because of the lower cost and higher availability of Mg^2+^ and Ca^2+^ as compared to Cs^+^. A subsequent work will assess Mg- and Ca-bentonite for one specific purpose: the CO_2_/CH_4_ separation in biogas upgrading by using a (vacuum-)pressure swing adsorption column.

## Supporting Information Available

Table of cation size and smectite *d*-spacing; schematic of the balance setup; description of the ex situ drying setup and procedure; CO_2_ adsorption on MMT using thermogravimetric analyzer; compilation of CO_2_ adsorption along the (de)hydration pathways; comparison of CO_2_ adsorption isotherms on MMT and bentonite (PDF)
