# Supplementary material for: CO2 Adsorption on Variably Hydrated Cation-Exchanged Montmorillonite-Rich Clays
Source: J Phys Chem C Nanomater Interfaces. 2025 Mar 26;129(14):6953–66. doi: 10.1021/acs.jpcc.4c07731 (PMC11998085; doi:10.1021/acs.jpcc.4c07731)
Supplement: Supplementary file 1 — jp4c07731_si_001.pdf [file jp4c07731_si_001.pdf]

Supporting information for: “CO_2 Adsorption on Variably Hydrated Cation-exchanged Montmorillonite-rich Clays”

Niels Mendel,[*,†] *Diana Sîrețanu,*[‡] *Igor Sîrețanu,*[†] *Derk W. F. (Wim) Brilman,*[‡] *and Frieder Mugele*[†]

[†]Physics of Complex Fluids, Faculty of Science and Technology, MESA+ Institute for Nanotechnology, University of Twente, P.O. Box 217, Enschede 7500 AE, The Netherlands

[‡]Sustainable Process Technology, Faculty of Science and Technology, University of Twente, P.O. Box 217, Enschede 7500 AE, The Netherlands

Corresponding author e-mail: n.mendel@utwente.nl

Contents:

**Table S1:**	cation size and smectite *d*-spacing (literature)
**Figure S1:**	a schematic of the balance setup
**Table S2:**	***ex situ* drying setup and procedure**
**Figure S2:**	CO_2 adsorption MMT dried under dry N_2 (thermogravimetric analyzer; isothermal)
**Figure S3:**	CO_2 adsorption MMT dried under dry N_2 (thermogravimetric analyzer; elevated temperatures)
**Figure S4:**	CO_2 adsorption as a function of sorbed H_2O – compilation of MMT and bentonite (de)hydration pathways
**Figure S5:**	CO_2 adsorption isotherms MMT
**Figure S6:**	comparison of CO_2 adsorption isotherms on MMT and bentonite

Table S1. Ranges of reported *d*-spacings for homoionic smectites and pyrophyllite in the absence of adsorbed molecules, and the ionic diameter of the respective cations.

Cation	Diam. (pm)	*d*-spacing (Å)	Smectite
-	-	9.2	Pyrophyllite[1,a]
Mg^{2+}	144[2]	9.77-10.8[b]	SWy[3,4]; SAz[5-7]; other[8]
Ca^{2+}	200[2]	9.55-10.0	SWy[3,4,9,10]; SAz[5,6,9,10]; other[11]
Na^{+}	204[2]	9.55-10.28	SWy[3,9,10,12-18]; SAz[9,10,12]; SHCa[19,20]; other[8,11,12,21-23]
Cs^{+}	334[2]	10.7-11.6	SWy[9,10,15,17,18]; SAz[5-7,9,10]; SHCa[19,20,24,25]; other[23,26]
MMA^{+}	434[27]	12.0	Other[28]
TMA^{+}	560[29]	13.4-13.9	SWy[9,10]; SAz[5-7,9,10]; SHCa[25]; other[28,30]

[a] Pyrophyllite is a charge-neutral smectite-like mineral (i.e., without interlayer cations); [b] Mg-smectites are notoriously difficult to dehydrate; some of the reported *d*-spacings may thus have been determined in the presence of tightly bound H_2O.[31]

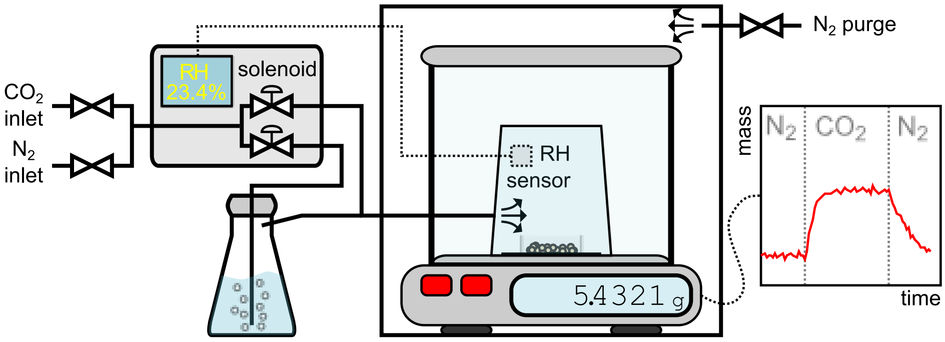

Figure S1. Schematic of the balance setup. Dry CO_2 or N_2 is fed to a humidistat. The humidistat directs part of this flow through a gas washing bottle to control the RH of the total inflow to a container enclosing the scale platform and sample. The environment of the balance is continuously purged using dry N_2 and the balance is read-out using a computer. The (homebuilt) humidistat is detailed in Ref. 32.

***Ex situ* drying**

This section discusses the setup and procedure used for the *ex situ* (pre-)drying under dry N_2 purge of the bentonite samples discussed in **Figure 7**b,f and **Figure 8**. The setup features a large plastic box with a volume of ~80 L that is continuously purged with dry N_2 with an input flow rate ~2.7 L min^{-1}. For the experiments presented in **Figure 7**b, initially 5 samples of Mg-bentonite powder and 5 samples of Ca-bentonite powder with a sample mass ~6.5 g each were placed inside the large box. Each bentonite sample was contained in a smaller cylindrical plastic container with a volume of 120 ml and diameter of ~5 cm. The top surface of this plastic container was open to the atmosphere within the larger box. At day 1, 2, 4, and 7, one sample of both Mg- and Ca-bentonite was removed from the setup and analyzed. The set of experiments above was repeated once more, starting with 6 samples of Mg- and Ca-bentonite each, and removing one sample of both Mg- and Ca-bentonite from the setup at day 14, 21, 55, 78, and 126. The total dry gas purge volume per unit sample mass is calculated on the basis of the *total* sample mass within the setup; see **Table S2**. The samples discussed in **Figure 8** were dried similarly and the total dry purge gas volume, calculated on the basis of the *total* sample mass within the drying setup, was such that the expected (CO_2) adsorption was near the plateau value (see **Figure 7**d,e; dried for several weeks).

Table S2. (Cumulative) dry gas purge volume during the *ex situ* drying, **Figure 7**b.

Day start	Day out	Number of samples in box	Dry purge gas volume (L g^{-1})	Cumulative dry purge gas volume (L g^{-1})
0	1	10	60	60
1	2	8	75	135
2	4	6	199	334
4	7	4	449	783
0	14	12	698	698
14	21	10	419	1117
21	41	8	1495	2612
41	55	6	1396	4008
55	78	4	3439	7447
78	126	2	14356	21803

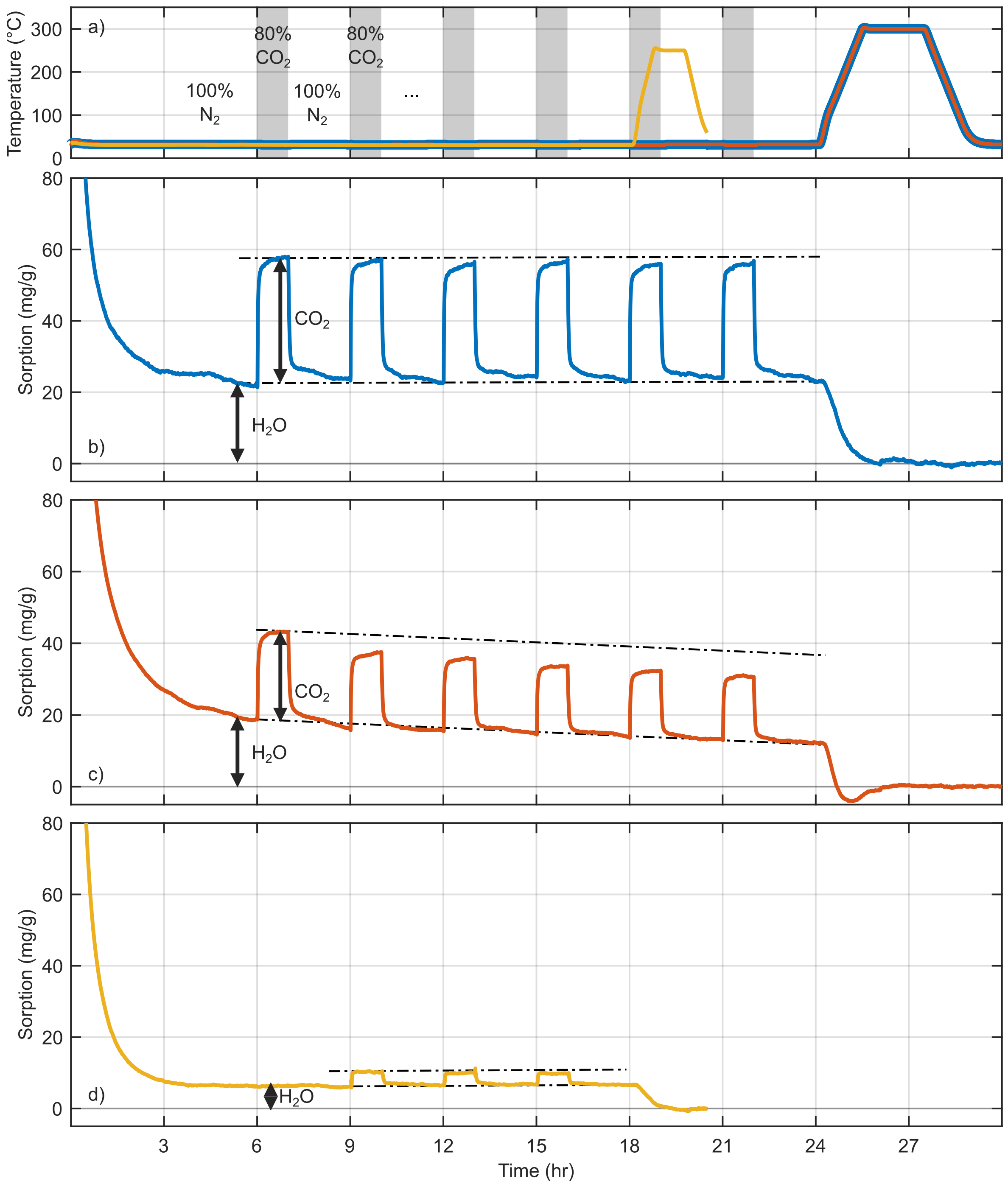

Figure S2. Desorption of H_2O and sorption of CO_2 as determined using the thermogravimetric analyzer on (b) Mg-MMT, (c) Ca-MMT, and (d) Na-MMT. The MMT was pre-hydrated over liquid H_2O, then dried under dry N_2, and the CO_2 sorption was determined regularly by switching to 80% CO_2 (balance N_2; see (a)). The total dry gas flow rate was 0.100 L min^{-1}. The dry sample mass was determined at the end of the experiment by heating, see (a).

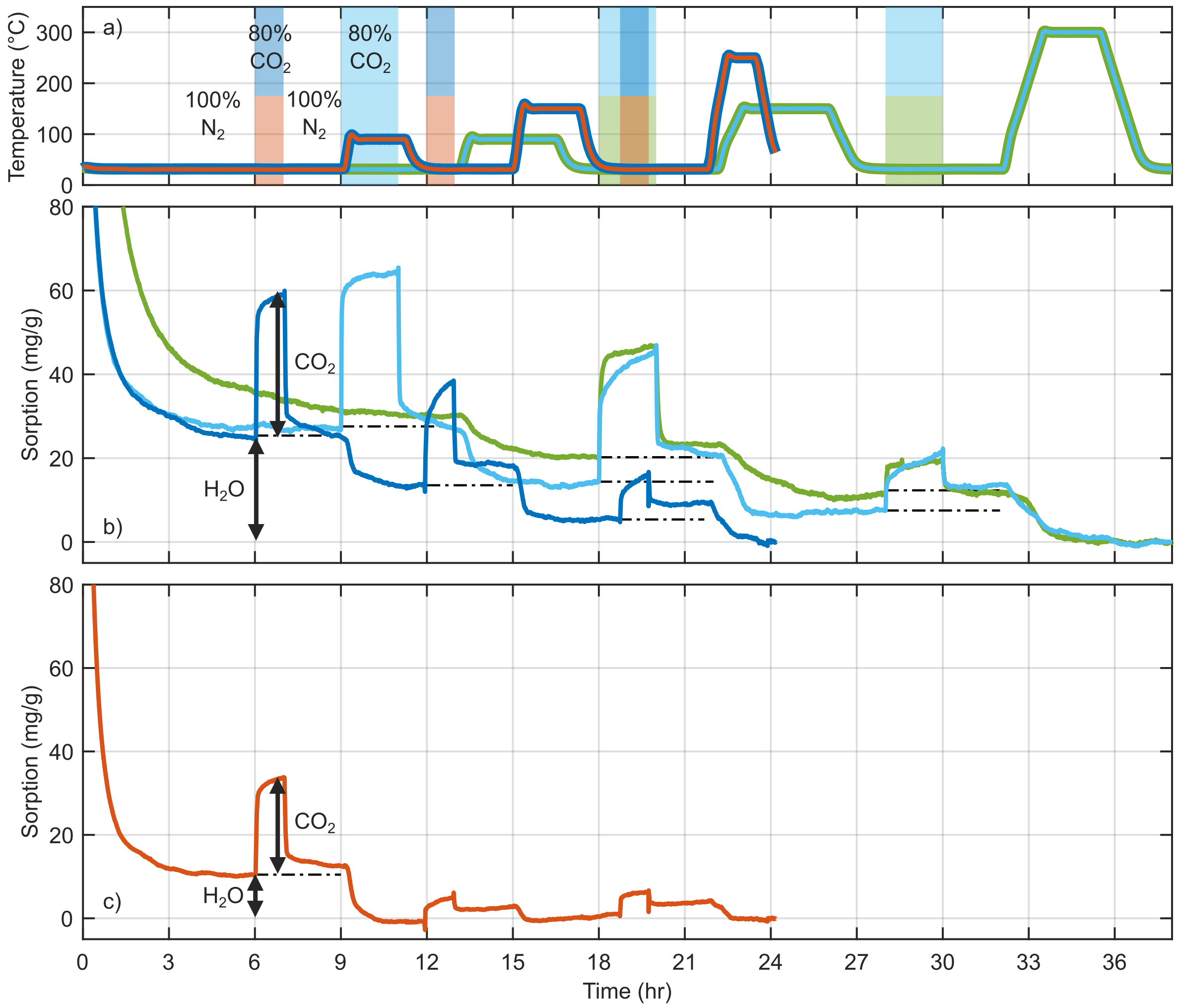

Figure S3. Desorption of H_2O and sorption of CO_2 as determined using the thermogravimetric analyzer on (b) Mg-MMT (three experiments) and (c) Ca-MMT. The MMT was pre-hydrated over liquid H_2O and dried under dry N_2 first at 30°C, then at 90°C, and finally at 150°C (see (a); colors correspond to the lines in (b) and (c)). In between, the CO_2 sorption was determined by switching to 80% CO_2 (balance N_2; see the shaded areas in (a); colors correspond to the lines in (b) and (c)). The total dry gas flow rate was 0.100 L min^{-1}. The dry sample mass was determined at the end of the experiment by heating to 250°C or 300°C, see (a).

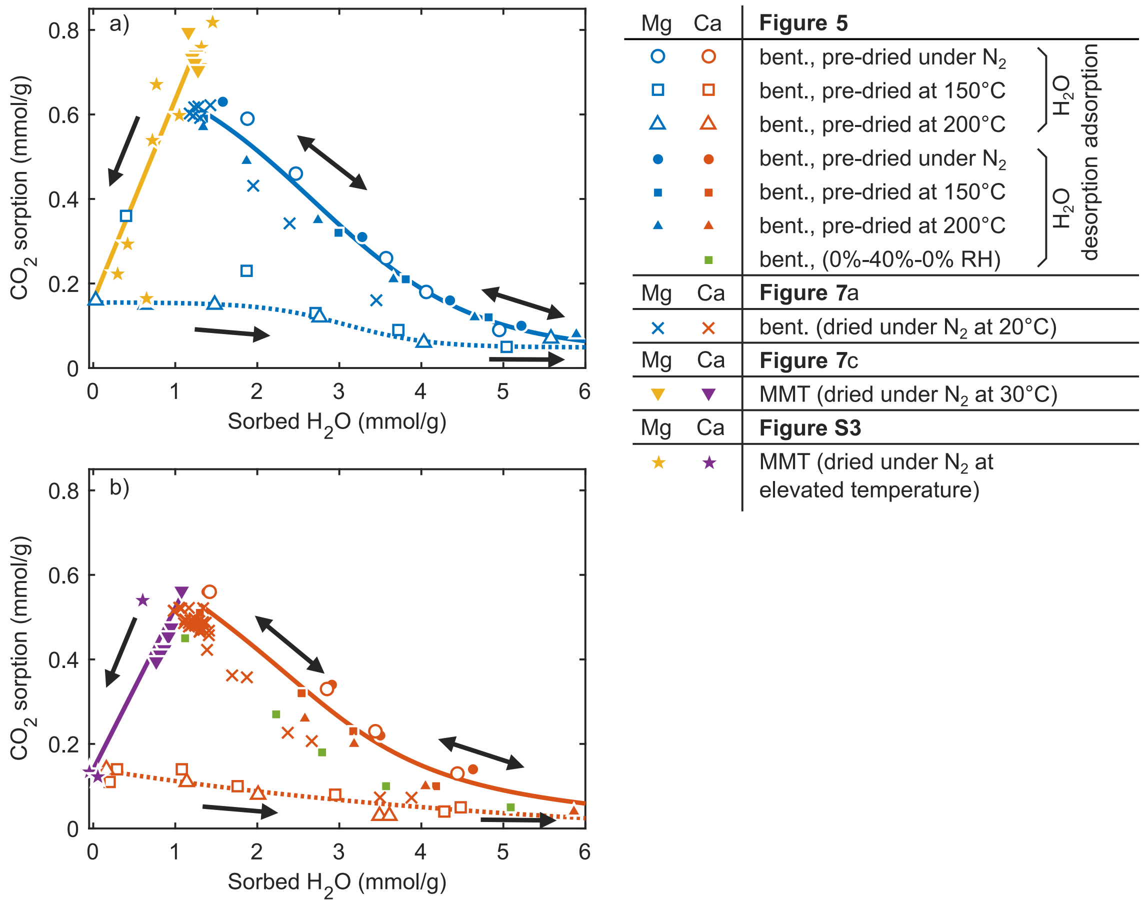

Figure S4. CO_2 sorption (at 1 bar, ~20°C; the sorption on the MMT samples is corrected for sample purity and the different experimental conditions) as a function of sorbed H_2O for (a) Mg-bentonite and -MMT and (b) Ca-bentonite and -MMT. The H_2O adsorption and desorption pathways are indicated by the black arrows. This Figure combines the results in **Figure 5**, **Figure 7**a,c, and **Figure S3** using the same symbols (see legend).

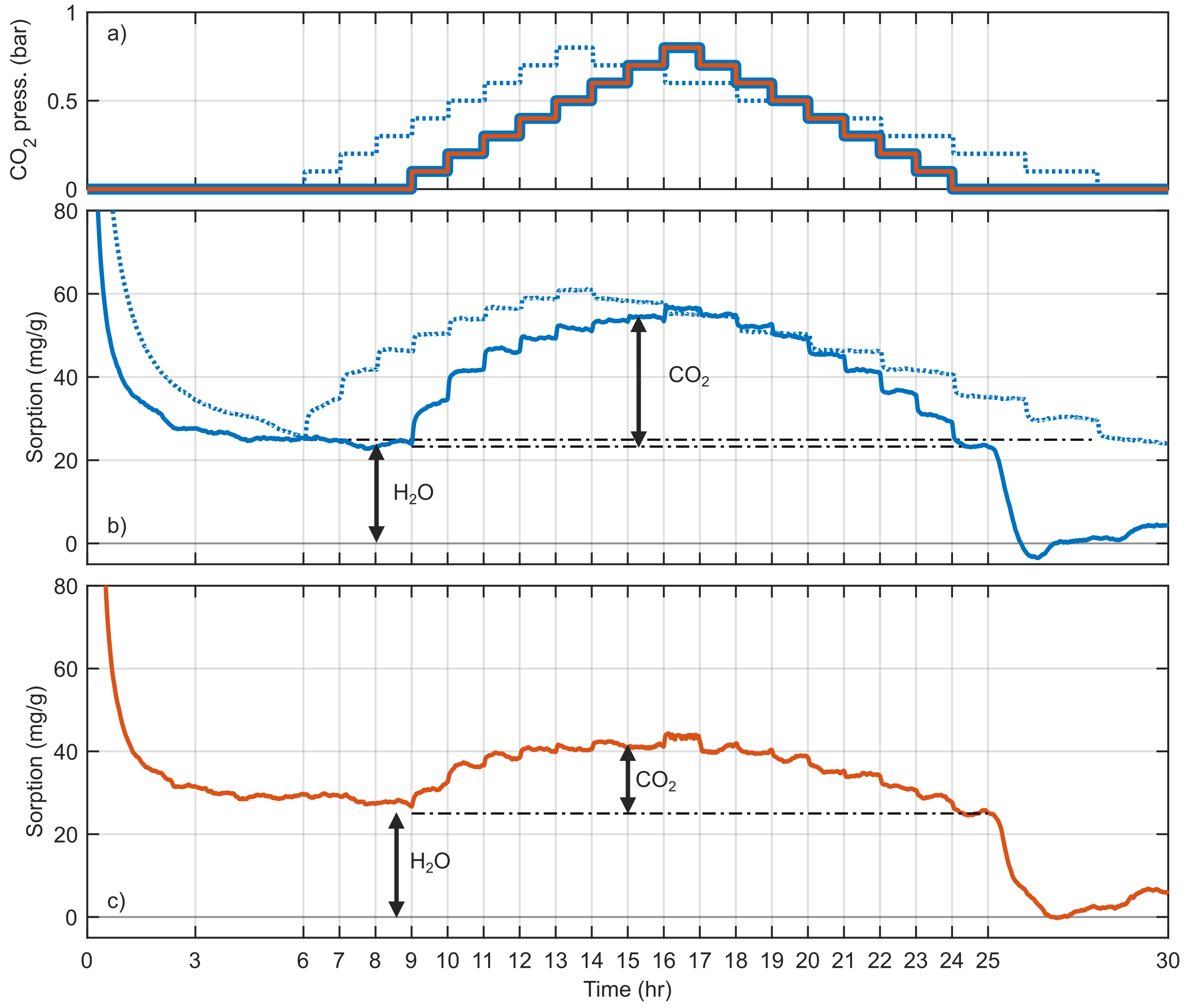

Figure S5. Desorption of H_2O and stepwise ad- and desorption of CO_2 at 30°C as determined using the thermogravimetric analyzer on (b) Mg-MMT (two experiments) and (c) Ca-MMT. The MMT was pre-hydrated over liquid H_2O and first dried under dry N_2. The CO_2 sorption was determined by the stepwise increase and decrease of the CO_2 partial pressure (0% – 80% – 0% CO_2; balance N_2; see (a), in which line style corresponds to the lines in (b) and (c)). The total dry gas flow rate was 0.100 L min^{-1}. The dry sample mass was determined at the end of the experiment by heating to ≥ 250°C, as in **Figures S2-S3** (the temperature evolution is not shown here for clarity).

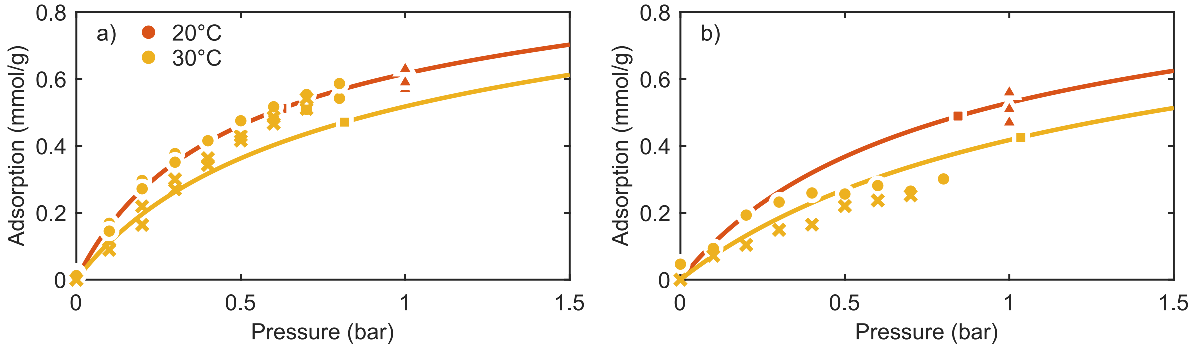

Figure S6. Sorption isotherms of CO_2 at 20℃ and 30℃ on (a) Mg-bentonite and -MMT and (b) Ca-bentonite and -MMT. This Figure combines the results in **Figure 4** (filled triangles; H_2O desorption, ~0% RH), **Figure 8** (filled squares; including the dual-site Langmuir fit, solid lines), and **Figure S5** (CO_2 adsorption: filled circles; CO_2 desorption: crosses). The sorption on the MMT samples in **Figure S5** is multiplied by 0.72 to account for the difference in sample purity. In general, the measured CO_2 adsorption (after accounting for the ~72% purity of the bentonite) agrees reasonably well between the three different experiments and confirms the rather large curvature of the CO_2 adsorption isotherms in the low (partial-)pressure domain. The MMT samples measured in the thermogravimetric analyzer (in particular Ca-MMT), however, are subject to a continuous desorption of H_2O that reduces the CO_2 adsorption capacity during the experiment, see also **Figure 7**c. In addition, the CO_2 sorption on Mg-MMT is ~15% larger than on Mg-bentonite, even after accounting for the different sample purity as is in agreement with **Figure 7**c.

References

1. Zelazny, L.W.; White, G.N., *The pyrophyllite–talc group.* Miner. Soil Environ., **1989**, *1*, 527-550.
2. Shannon, R.D., *Revised effective ionic radii and systematic studies of interatomic distances in halides and chalcogenides.* Acta Crystallogr., Sect. A: Cryst. Phys., Diffr., Theor. Gen. Crystallogr., **1976**, *32*, 751-767.
3. Dios Cancela, G.; Huertas, F.J.; Romero Taboada, E.; Sánchez-Rasero, F.; Hernández Laguna, A., *Adsorption of water vapor by homoionic montmorillonites. Heats of adsorption and desorption.* J. Colloid Interface Sci., **1997**, *185*, 343-354.
4. Cases, J.-M.; Bérend, I.; François, M.; Uriot, L.P.; Michot, L.J.; Thomas, F., *Mechanism of Adsorption and Desorption of Water Vapor by Homoionic Montmorillonite: 3. The Mg^{2+}, Ca^{2+}, Sr^{2+} and Ba^{2+} Exchanged Forms.* Clays Clay Miner., **1997**, *45*, 8-22.
5. Ziemiański, P.P.; Derkowski, A.; Szczurowski, J.; Kozieł, M., *The structural versus textural control on the methane sorption capacity of clay minerals.* Int. J. Coal Geol., **2020**, *224*, 103483.
6. Ziemiański, P.P.; Derkowski, A.; Szczerba, M.; Guggenheim, S., *Smectite crystallite swelling under high pressure of methane.* J. Phys. Chem. C, **2021**, *125*, 7598-7610.
7. Ziemiański, P.P.; Derkowski, A., *Structural and textural control of high-pressure hydrogen adsorption on expandable and non-expandable clay minerals in geologic conditions.* Int. J. Hydrogen Energy, **2022**, *47*, 28794-28805.
8. Grekov, D.I.; Suzuki-Muresan, T.; Kalinichev, A.G.; Pré, P.; Grambow, B., *Thermodynamic data of adsorption reveal the entry of CH_4 and CO_2 in a smectite clay interlayer.* Phys. Chem. Chem. Phys., **2020**, *22*, 16727-16733.
9. Rutherford, D.W.; Chiou, C.T.; Eberl, D.D., *Effects of exchanged cation on the microporosity of montmorillonite.* Clays Clay Miner., **1997**, *45*, 534-543.
10. Chiou, C.T.; Rutherford, D.W., *Effects of exchanged cation and layer charge on the sorption of water and EGME vapors on montmorillonite clays.* Clays Clay Miner., **1997**, *45*, 867-880.
11. Holmboe, M.; Wold, S.; Jonsson, M., *Porosity investigation of compacted bentonite using XRD profile modeling.* J. Contam. Hydrol., **2012**, *128*, 19-32.
12. Sato, T.; Watanabe, T.; Otsuka, R., *Effects of layer charge, charge location, and energy change on expansion properties of dioctahedral smectites.* Clays Clay Miner., **1992**, *40*, 103-113.
13. Giesting, P.; Guggenheim, S.; Koster van Groos, A.F.; Busch, A., *Interaction of carbon dioxide with Na-exchanged montmorillonite at pressures to 640 bars: Implications for CO_2 sequestration.* Int. J. Greenhouse Gas Control, **2012**, *8*, 73-81.
14. Cases, J.M.; Bérend, I.; Besson, G.; Francois, M.; Uriot, J.P.; Thomas, F.; Poirier, J.E., *Mechanism of adsorption and desorption of water vapor by homoionic montmorillonite. 1. The sodium-exchanged form.* Langmuir, **1992**, *8*, 2730-2739.
15. Bérend, I.; Cases, J.-M.; François, M.; Uriot, J.-P.; Michot, L.; Masion, A.; Thomas, F., *Mechanism of adsorption and desorption of water vapor by homoionic montmorillonites: 2. The Li^+ Na^+, K^+, Rb^+ and Cs^+-exchanged forms.* Clays Clay Miner., **1995**, *43*, 324-336.
16. Schaef, H.T.*; et al.*, *Competitive sorption of CO_2 and H_2O in 2:1 layer phyllosilicates.* Geochim. Cosmochim. Acta, **2015**, *161*, 248-257.
17. Schaef, H.T.*; et al.*, *Tipping Point for Expansion of Layered Aluminosilicates in Weakly Polar Solvents: Supercritical CO_2.* ACS Appl. Mater. Interfaces, **2017**, *9*, 36783-36791.

18. Loring, J.S.*; et al.*, *Synergistic Coupling of CO_2 and H_2O during Expansion of Clays in Supercritical CO_2–CH_4 Fluid Mixtures.* Environ. Sci. Technol., **2021**, *55*, 11192-11203.
19. Loganathan, N.; Bowers, G.M.; Yazaydin, A.O.; Schaef, H.T.; Loring, J.S.; Kalinichev, A.G.; Kirkpatrick, R.J., *Clay Swelling in Dry Supercritical Carbon Dioxide: Effects of Interlayer Cations on the Structure, Dynamics, and Energetics of CO_2 Intercalation Probed by XRD, NMR, and GCMD Simulations.* J. Phys. Chem. C, **2018**, *122*, 4391-4402.
20. Bowers, G.M.; Loring, J.S.; Schaef, H.T.; Walter, E.D.; Burton, S.D.; Hoyt, D.W.; Cunniff, S.S.; Loganathan, N.; Kirkpatrick, R.J., *Interaction of hydrocarbons with clays under reservoir conditions: in situ infrared and nuclear magnetic resonance spectroscopy and X-ray diffraction for expandable clays with variably wet supercritical methane.* ACS Earth Space Chem., **2018**, *2*, 640-652.
21. Morodome, S.; Kawamura, K., *Swelling behavior of Na-and Ca-montmorillonite up to 150°C by in situ X-ray diffraction experiments.* Clays Clay Miner., **2009**, *57*, 150-160.
22. Ilton, E.S.; Schaef, H.T.; Qafoku, O.; Rosso, K.M.; Felmy, A.R., *In situ X-ray diffraction study of Na^+ saturated montmorillonite exposed to variably wet super critical CO_2.* Environ. Sci. Technol., **2012**, *46*, 4241-4248.
23. Hunvik, K.W.B.*; et al.*, *Intercalation of CO_2 selected by type of interlayer cation in dried synthetic hectorite.* Langmuir, **2023**, *39*, 4895-4903.
24. Bowers, G.M.; Schaef, H.T.; Loring, J.S.; Hoyt, D.W.; Burton, S.D.; Walter, E.D.; Kirkpatrick, R.J., *Role of Cations in CO_2 Adsorption, Dynamics, and Hydration in Smectite Clays under in Situ Supercritical CO_2 Conditions.* J. Phys. Chem. C, **2017**, *121*, 577-592.
25. Bowers, G.M.; Loring, J.S.; Walter, E.D.; Burton, S.D.; Bowden, M.E.; Hoyt, D.W.; Arey, B.; Larsen IV, R.K.; Kirkpatrick, R.J., *Influence of smectite structure and hydration on supercritical methane binding and dynamics in smectite pores.* J. Phys. Chem. C, **2019**, *123*, 29231-29244.
26. Morodome, S.; Kawamura, K., *In situ X-ray diffraction study of the swelling of montmorillonite as affected by exchangeable cations and temperature.* Clays Clay Miner., **2011**, *59*, 165-175.
27. Kieslich, G.; Sun, S.; Cheetham, A.K., *Solid-state principles applied to organic–inorganic perovskites: new tricks for an old dog.* Chem. Sci., **2014**, *5*, 4712-4715.
28. Barrer, R.M.; Reay, J.S.S., *Sorption and intercalation by methyl-ammonium montmorillonites.* Trans. Faraday Soc., **1957**, *53*, 1253-1261.
29. Marcus, Y., *Thermodynamics of solvation of ions. Part 5.—Gibbs free energy of hydration at 298.15 K.* J. Chem. Soc., Faraday Trans., **1991**, *87*, 2995-2999.
30. Barrer, R.M.; MacLeod, D.M., *Activation of montmorillonite by ion exchange and sorption complexes of tetra-alkyl ammonium montmorillonites.* Trans. Faraday Soc., **1955**, *51*, 1290-1300.
31. Kuligiewicz, A.; Derkowski, A., *Tightly bound water in smectites.* Am. Mineral., **2017**, *102*, 1073-1090.
32. Veldscholte, L.B.; de Beer, S., *OpenHumidistat: Humidity-controlled experiments for everyone.* HardwareX, **2022**, *11*, e00288.
